# Introduced and invasive cactus species: a global review

**DOI:** 10.1093/aobpla/plu078

**Published:** 2014-12-03

**Authors:** Ana Novoa, Johannes J. Le Roux, Mark P. Robertson, John R.U. Wilson, David M. Richardson

**Affiliations:** 1 Centre for Invasion Biology, Department of Botany and Zoology, Stellenbosch University, Matieland 7602, South Africa; 2 Centre for Invasion Biology, Department of Zoology and Entomology, University of Pretoria, Pretoria 0002, South Africa; 3 Invasive Species Programme, South African National Biodiversity Institute, Kirstenbosch Research Centre, Claremont 7735, South Africa

**Keywords:** Biological invasions, cactus invasions, climate suitability, introduction pathways, invasion debt, invasive species, phylogenetic signal.

## Abstract

Understanding which species are introduced and become invasive, and why, are central questions in invasion science. Comparative studies on model taxa have provided important insights, but much more needs to be done to unravel the context dependencies of these findings. The cactus family (Cactaceae), one of the most popular horticultural plant groups, is an interesting case study. Hundreds of cactus species have been introduced outside their native ranges; a few of them are among the most damaging invasive plant species in the world. We reviewed the drivers of introductions and invasions in the family and seek insights that can be used to minimize future risks. We compiled a list of species in the family and determined which have been recorded as invasive. We also mapped current global distributions and modelled the potential global distributions based on distribution data of known invasive taxa. Finally, we identified whether invasiveness is phylogenetically clustered for cacti and whether particular traits are correlated with invasiveness. Only 57 of the 1922 cactus species recognized in this treatment have been recorded as invasive. There are three invasion hotspots: South Africa (35 invasive species recorded), Australia (26 species) and Spain (24 species). However, there are large areas of the world with climates suitable for cacti that are at risk of future invasion—in particular, parts of China, eastern Asia and central Africa. The invasive taxa represent an interesting subset of the total species pool. There is a significant phylogenetic signal: invasive species occur in 2 of the 3 major phylogenetic clades and in 13 of the 130 genera. This phylogenetic signal is not driven by human preference, i.e. horticultural trade, but all invasive species are from 5 of the 12 cactus growth forms. Finally, invasive species tend to have significantly larger native ranges than non-invasive species, and none of the invasive species are of conservation concern in their native range. These results suggest fairly robust correlates of invasiveness that can be used for proactive management and risk assessments.

## Abstract (precis)

Understanding which species are introduced and become invasive are central questions in invasion science. In this sense, the cactus family is an interesting case study. Only 57 of the 1922 cactus species are currently recorded as invasive. There are three invasion hotspots: South Africa, Australia, and Spain. However, we identified large areas of the world with suitable climates. The invasive taxa represent an interesting subset of the total pool: they occur in two of the three major phylogenetic clades and in 13 of the 130 cactus genera, they possess five of the 12 cactus growth forms, and they tend to have larger native ranges.

## Introduction

The increased movement of humans around the world has facilitated the intentional and accidental transportation of species far from their native ranges, often in a manner that can facilitate invasions (Wilson *et al.* 2009). Many of these introduced organisms have notable benefits to humans, but some have undesirable impacts that can result in substantial monetary costs and/or alterations to entire ecosystems and social systems (McNeely 2006; Kumschick *et al.* 2012). Government departments, non-governmental organizations, extension services, environmental managers, conservationists and scientists are all facing escalating pressure to address and resolve a diversity of problems posed by invasive alien species (Hulme 2006).

Much work has recently focussed on reviewing the invasive performance of particular genera or closely related groups of organisms in different situations around the world (e.g. Richardson *et al.* 2011; Moodley *et al.* 2013; Potgieter *et al.* 2013; Shackleton *et al.* 2014). Such studies aim to update knowledge on the global occurrence and potential range of these taxa and to understand the complex drivers of human-mediated introductions and invasions. The findings of such studies are important for developing protocols for preventing risky species introductions and for managing species that may become or have already become invasive (Simberloff *et al.* 2009). However, more comparative studies are needed to improve our understanding of the full suite of interacting factors that influence invasions and to unravel the context dependencies of insights that emerge from particular studies (Kueffer *et al.* 2013). It is important to consider whether such comparisons yield broad generalities or whether generalizations apply only to a subset of taxa.

The cactus family (Cactaceae; ‘cacti’) is an interesting case study. Cacti are a conspicuous component of the arid regions of the New World and represent one of the world's most spectacular desert radiations (Edwards *et al.* 2005). The family is distributed from southern Patagonia in Argentina and Chile to Alberta and British Columbia in Canada (Edwards *et al.* 2005), with the only exception being *Rhipsalis baccifera* (mistletoe cactus), which is thought to have originated in tropical Americas, but was apparently dispersed across the Atlantic Ocean by birds, reaching southern Africa, Madagascar and Sri Lanka (Rebman and Pinkava 2001).

Cacti are among the first plants that were brought back from the Americas by European explorers in the 15th century (Howard and Touw 1981) and soon became common in European collections and gardens (Anderson 2001). The trade in horticultural cacti has developed over the years into a substantial industry and is responsible for the intercontinental spread of many species (Walters *et al.* 2011). One of the earliest reasons for introduction, however, was for use as drought-tolerant crops and for hedging, with *Opuntia ficus-indica* (L.) Mill. being by far the most utilized (Walters *et al.* 2011). In an attempt to minimize the risks of global climate change, land degradation and diminishing food security, the Food and Agricultural Organization has revived the interest in cactus cultivation for agricultural purposes in developing countries (Nefzaoui 2007). As a result of these human-mediated introductions, cactus species can be found all over the world, and several members of the family are among the most important alien species worldwide (Weber 2003).

Studies of cactus invasions have shed light on crucial aspects of plant invasion ecology, e.g. the interaction of invasive plants with seed dispersers (Foxcroft and Rejmánek 2007; Padrón *et al.* 2011), the role of propagule pressure in driving invasions (Foxcroft *et al.* 2004) and the role of herbivores in regulating some plant populations, with particularly striking examples from classical biological control (Zimmermann *et al.* 2004; Paterson *et al.* 2011). These studies have tended to focus on the genera *Opuntia* and *Cylindropuntia*, which contain most of the widely introduced, cultivated and invasive species in the family. However, hundreds of new cactus species are now being introduced all over the world, and many of them are becoming naturalized or invasive. For example, *Cereus hexagonus* was included in a national list of regulated invasive species for the first time in South Africa in 2014.

The current global distribution of the cactus family is being radically changed by humans, and no attempt has been made to assess the status of each species in terms of invasion or risk thereof. Consequently, a broad global assessment of the determinants of invasiveness of the family Cactaceae is an important requirement for the formulation of control strategies. Moreover, reviewing the invasive performance of this family around the world may uncover new patterns, processes and invasion risks not seen in better-studied model groups.

This paper aims specifically to (i) compile a list of species in the family Cactaceae, (ii) determine their current native and invasive ranges, and (iii) determine the potential future ranges of invasive taxa. Using these lists we aim to answer the questions: (iv) how have cactus species been used inside and outside their native range?; and (vi) are any traits correlated with invasiveness in the family?

## Methods

### Defining a cactus

**Figure 1.**
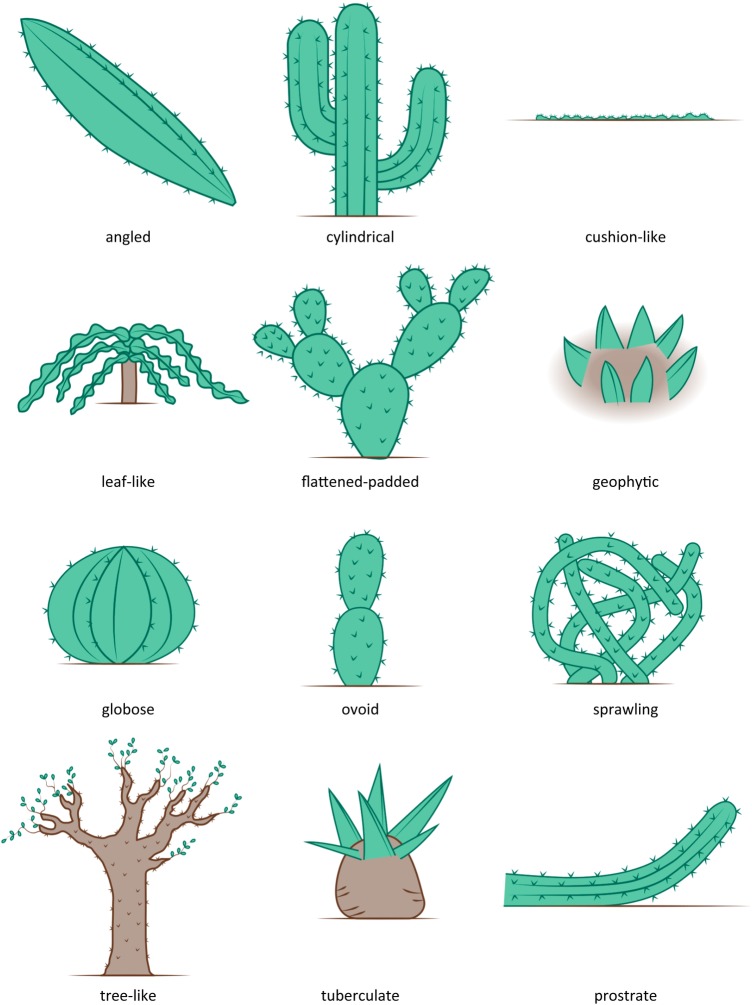
The 12 growth-form categories of the family Cactaceae considered in this paper.

Most taxa in the family Cactaceae are succulents with large, leafless, long-living, fleshy stems of different shapes and sizes that often contain clusters of spines which arise from areoles (Benson 1979, 1982; Eggli 1993). Areoles—highly specialized axillary or lateral buds or short shoots or branches—are unique to the family (Mauseth 1983; Gibson and Nobel 1986). However, cacti come in a wide range of growth forms (Fig. 1). Succulent plants in other families are often mistakenly called ‘cacti’ on nursery labels and in popular publications. While it is usually easy to distinguish cacti from other succulents, some taxa look very cactus-like due to convergent evolution, e.g. many species in the genus *Euphorbia* of the family Euphorbiaceae (Anderson 2001).

### Species list and invasive status

While the alpha taxonomy of the Cactaceae is well known, and the clade is clearly a recent monophyletic radiation, a stabilized nomenclature has not yet been achieved (Hunt and Taylor 1986, 1990; Nobel 2002). This nomenclature instability can be attributed to inconsistencies in previous taxonomies and to the generally poor representation of cacti in herbarium collections [their succulence and spines make them difficult to collect and curate (Walters *et al.* 2011)]. The International Organization for Succulent Studies recently attempted to revise the taxonomy of the Cactaceae (Hunt *et al.* 2006), but concluded that this task is far from complete.

In this paper, we base our list on the classification system developed by the International Cactaceae Systematics Group and used by David Hunt in compiling both editions of the Convention on International Trade in Endangered Species of Wild Fauna and Flora's CITES Cactaceae Checklist (1992, 1999). This classification has been adopted by various sources (Walters 1989; Kubitzki *et al.* 1993; Anderson 2001). We updated the list to include 109 additional species, for which, since 2001, either an International Union for Conservation of Nature and Natural Resources red list assessment has been made (http://www.iucnredlist.org/), or a new description has been published in the scientific literature. None of these additional 109 species are recorded as invasive.

Information on the current distribution and invasive status was gathered from many sources **[see Supporting Information]**. Each source uses different criteria for categorizing alien species. To record the invasive status of the family Cactaceae, our list includes only cactus species where there is clear documented evidence of an invasion as per the definition in Richardson *et al.* (2000), i.e. plants spreading over considerable distances from original planting or introduction sites.

### Potential distribution

Invasiveness elsewhere combined with broad-scale climatic matching are the most widely used predictors of future invasiveness of introduced species (Rejmánek *et al.* 2005; Richardson *et al.* 2011; Petitpierre *et al.* 2012). Determining which global areas are climatically suitable for invasive species establishment may aid future management efforts and/or preventative measures. We therefore used bioclimatic models for invasive cacti to predict potential suitable ranges for individual species on a global scale. To do this, we compiled a dataset of occurrence records for invasive cactus species from several different sources (e.g. Base de datos de ejemplares de cactáceas de Norte y Centro América, Herbario Virtual da Flora e dos Fungos, Intermountain Regional Herbarium Network, Global Biodiversity Information Facility, Instituto de Biologia de la UNAM, Southwest Environmental Information Network, Oregon Flora Project, San Diego Natural History museum and CalFlora). For each species, the records were plotted on a map and climatic space as defined by values of annual mean temperature and annual precipitation extracted from 10-min resolution WorldClim bioclimatic variables. A thorough data cleaning procedure was followed using the biogeo package in R (M. P. Robertson *et al.,* in preparation): tests were performed on obvious outliers to determine whether the *x*- and *y*-coordinates had accidentally been transposed or whether incorrect signs were used; records that were plotted in the ocean but directly adjacent to a coastal grid cell were assigned to that closest terrestrial grid cell; and low-precision records were removed, e.g. when only degrees were available for the coordinate. Outliers in the environmental space were queried to identify where they lay in geographical space to identify any errors missed during the initial data cleaning, and removed or rectified if a particular cause of the error could be determined.

Species distribution models were produced for each species using a simple envelope approach (implemented in R) which is equivalent to BIOCLIM's marginal envelope (Pearson and Dawson 2003). The following predictor variables were used: maximum temperature of warmest month, minimum temperature of coldest month, precipitation of wettest quarter, precipitation of driest quarter, precipitation of warmest quarter and precipitation of coldest quarter. These variables were obtained from WorldClim at a 10-min spatial resolution (Hijmans *et al.* 2005) and were selected based on their success at predicting potential global distributions for other model invasive taxa (Richardson *et al.* 2011).

Two sets of models were produced using different approaches. For the first approach, models were calibrated using native range records only. These models were then evaluated using invasive range records, where these were available. Sensitivity values (Fielding and Bell 1997) were calculated for each model based on the number of invasive range records that were predicted as present or absent by the model. Sensitivity values range between zero and one, where values close to one indicate low omission error (Fielding and Bell 1997). For the second approach, models were calibrated using all available records for the species (i.e. native and invasive range records) without evaluation of sensitivity. For both approaches we produced models for species that had five or more native range records. Duplicate records per 10-min cell were removed.

Maps of potential species richness were produced by adding the maps of potential distribution for each of the two approaches.

### Reasons for introduction and dissemination

Information on human uses of cacti both in their native and introduced ranges were extracted from many sources **[see Supporting Information]**. Five broad human-use categories were defined: (i) ornamental (horticulture), (ii) food or fodder (i.e. for humans or livestock), (iii) medicinal, (iv) hedging and (v) other (e.g. furniture or religious). Not all the species with a defined use in the native range are introduced to other areas of the world for the same reasons. To assess how the number of introduced species differs between uses, we compared the proportion of introduced and non-introduced species in each use category with that of species in other use categories (using a Fisher Test in R).

### Correlates of invasiveness

A useful first step to improve our understanding of invasiveness is to identify the traits correlated with invasiveness (Pyšek and Richardson 2007). Here, we looked at phylogeny, taxonomy (at the genus level), growth form and metrics of native range size.

For phylogenetic reconstruction we collated genetic data for the maturase K (matK) gene region for representative taxa of all Cactaceae genera with available data in the GenBank online repository (http://ncbi.nlm.nih.gov). DNA sequence data were aligned in BioEdit version 7.0.5.3 (Hall 1999) and manually edited. Because of differences in sequence lengths for different taxa we trimmed flanking regions to avoid excessive missing data. Our final dataset comprised 103 genera within Cactaceae. Phylogenetic relationships were estimated using Bayesian search criteria with parameter estimates obtained from the program jModelTest v2.1.3 (GTR + I + G; Darriba *et al.* 2012) in MrBayes 3.1.2 (Ronquist and Huelsenbeck 2003). For both datasets, MrBayes was run for 2 000 000 generations and trees sampled every 1000 generations. Nodal support for the retrieved tree topology was determined as posterior probabilities in MrBayes. To determine whether invasiveness within Cactaceae has a phylogenetic signal, we compared trait change (proportion of invasive taxa within a genus) with a null hypothesis of Brownian motion using Blomberg's *K* statistic (Blomberg *et al.* 2003). Similarly, we used Pagel's lambda (*λ*) statistic to determine the extent to which branch length transformation explains the distribution of trait states (proportion of invasive taxa/genus) at the tips of a phylogeny (Pagel 1999). Both tests are implemented in the function phylosig.R from the phytools package (Revell 2012).

To assess how invasiveness differs at the genus level, we compared the number of invasive and non-invasive species in each genus with that in the rest of the family using a Fisher test in R.

Based on information extracted from all sources, we also obtained information on the growth form of each species. There are different classifications in the literature (e.g. Barthlott and Hunt 1993; Anderson 2001; López and Valdivia 2007; Ortega-Baes *et al.* 2010; Hernández-Hernández *et al.* 2011). Here, following discussions in Anderson (2001) and Hernández-Hernández *et al.* (2011), we recognize 12 types: angled, cylindrical, cushion-like, leaf-like, flattened-padded, geophytic, globose, ovoid, sprawling tree-like, tuberculate and prostrate growth forms (Fig. 1).

Available data on native range size are inadequate for an analysis of the relationship between native range size and invasive status for the whole family. We were, however, able to analyse the relationship between native range size and invasiveness for the genus *Opuntia*, because species in the genus have been widely introduced and disseminated around the world, there are many invasive and non-invasive taxa, and there are comparatively good data on native range size for this genus (Dean and Milton 2000; Erre *et al.* 2009; Padrón *et al.* 2011; Lloyd and Reeves 2014). We compared the latitudinal ranges of invasive and non-invasive *Opuntia* species using a Student's *t*-test in R. We also looked at Cactaceae listed on the IUCN Red List of Threatened Species (Rodrigues *et al.* 2006). For Cactaceae, most of the species considered at risk were so due to a small native range size (http://www.iucnredlist.org/), and therefore, in this case, the list provides a rough proxy for native range size (as well as giving some indication of population trends).

## Results

### Species list and invasive status

The list of cacti assembled for this paper comprises 1922 species from 130 genera **[see Supporting Information]**. Genera differed widely in species richness, with several speciose genera [*Opuntia* (193 species), *Echinopsis* (133 species) and *Mammillaria* (171 species)] and 35 monotypic genera. Of the 1922 species we could definitively classify only 57 species as invasive.

**Figure 2.**
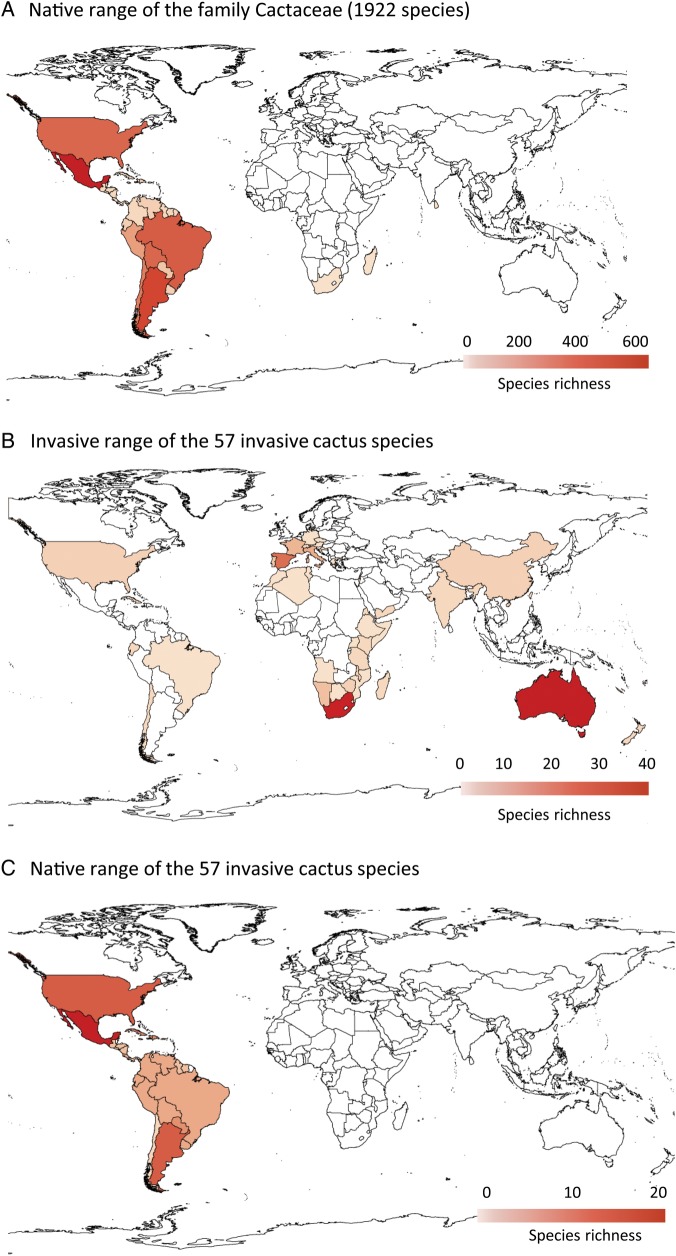
Cactus species richness across the native (A) and invasive range (B) as well as the native distribution of invasive cacti (C). Shading indicates the number of taxa per country. Lighter colors correspond to less taxa.

The currently available distribution data are only adequate for a country-level analysis (see Fig. 2). These analyses show Mexico as the main ‘hot spot’ of native cactus diversity (Fig. 2A). Three countries had notably more invasive taxa than the rest—Australia (39), South Africa (35 species) and Spain (24)—while other countries had at most 13 (Fig. 2B, Table 1). The most widespread invasive species is *O. ficus-indica* (22 different countries), with other species invading 15 or fewer countries (Table 1). Unsurprisingly, the origin of most of these invasive species is also Mexico (Fig. 2C).

**Table 1.** Distribution of invasive Cactaceae species outside their native range. Data were compiled from a range of sources **[see Supporting Information]**.

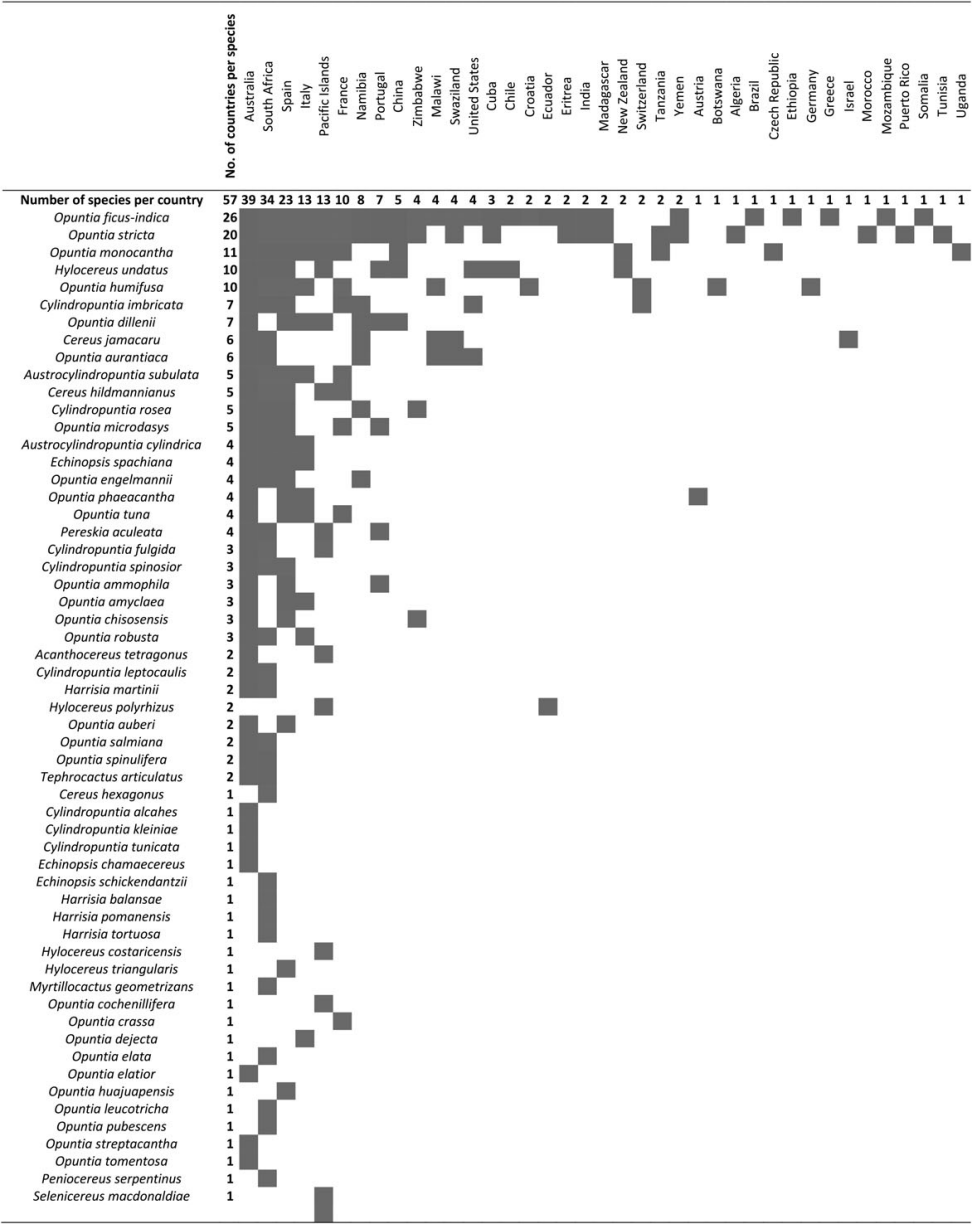

### Potential distribution

**Figure 3.**
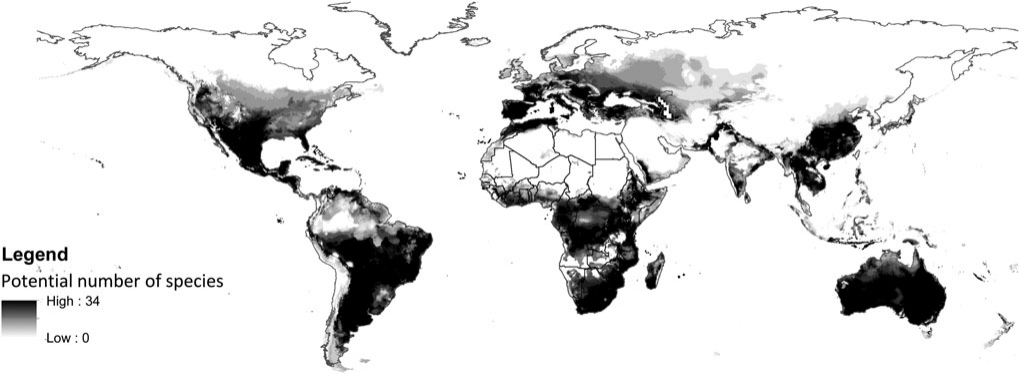
Potential species richness based on all available records (native and invasive records) of 39 invasive cactus species.

We examined potential invasive distributions for only 39 of the invasive species **[see Supporting Information]** as none of the remaining 18 species had enough (i.e. five or more) records in their native ranges of sufficient accuracy. The median number of records per species modeled was 128 for the native range and 124 for the invasive range. A large variation in sensitivity values was obtained. We found no significant differences between the projected species richness maps for the two modelling approaches, i.e. using native range occurrence records only or native and invasive occurrence records (Fig. 3). The main known areas of invasion (Australia, South Africa and Spain) were indicated as suitable, but there were also substantial regions that are suitable in central Africa, China and south-eastern Asia.

### Reasons for introduction and dissemination

A quarter of species recorded as being used for ornamentation in their native ranges have been introduced elsewhere (Table 2). In contrast, only a seventh of the cactus species used in their native ranges for food or fodder have been introduced elsewhere, and we found no official records of species having been introduced for medicinal or other purposes (though *Lophophora williamsii* and *Echinopsis pachanoi* have been introduced worldwide for their psycho-active uses). As an illustration of the worldwide popularity of cacti as horticultural species, we give some examples of international cactus and succulent journals and societies **[see Supporting Information]**.

**Table 2.** The number of species inside and outside their native range across human uses. Note that one species can be included in more than one use category. Significance levels were determined by comparing the number of introduced vs. number of non-introduced species for any category to all other taxa using Fisher's exact test. Confidence intervals were determined for the percentage of introduced or non-introduced based on an assumption of binomial errors. Other uses include minor uses such as water source.

	Ornamental	Food	Medicinal	Hedging	Other
Native range	837	261	345	15	10
Non native range	250	45	0	5	0
Percentage (95 % CIs)	23.0 % (20.5–25.6)	14.7 % (10.9–19.2)	0 % (0–10.3)	25.0 % (8.7–49.1)	0 % (0–30.8)
Significant	*P* < 0.01	*P* < 0.01	*P* < 0.01	*P* < 0.58	*P* < 0.23

### Correlates of invasiveness

**Figure 4.**
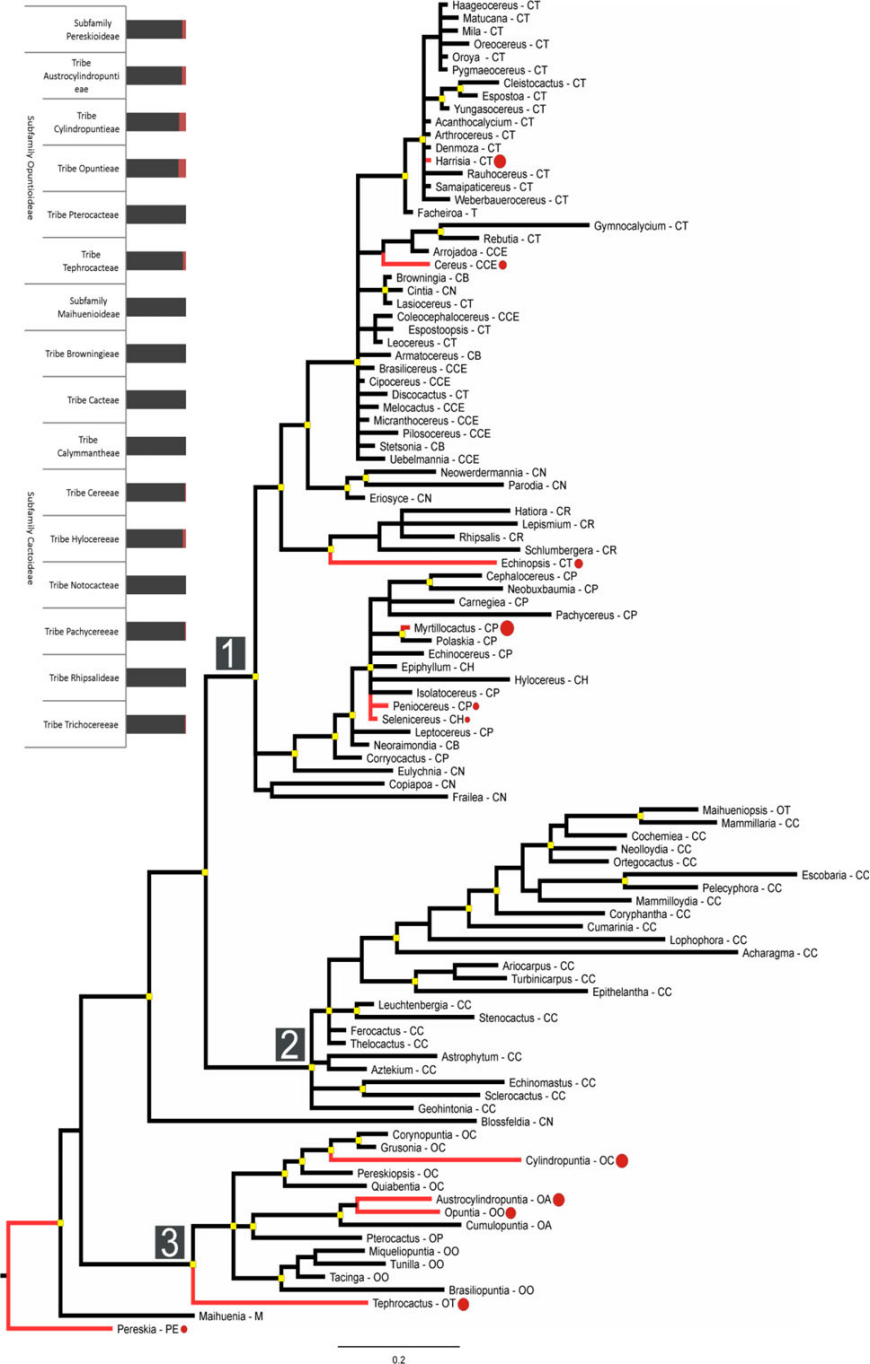
Bayesian phylogeny based on matK DNA sequence data illustrating phylogenetic relationships among genera within Cactaceae. The aligned matK matrix contained 1354 characters and required 65 gaps (indels), ranging from 1 to 74 characters in size. Overall, the phylogeny yielded well-resolved relationships among all genera included. High nodal support (posterior probabilities >0.9) is indicated at nodes by yellow boxes. Genera with invasive taxa are indicated as red branches where red circles are proportional to the percentage of invasive taxa within each genus. Scale bar = number of substitutions/site. The three main clades are indicated under the numbers 1, 2 and 3. CB: Subfamily Cactoideae, Tribe Browningieae; CC: Subfamily Cactoideae, Tribe Cacteae; CCE: Subfamily Cactoideae, Tribe Cereeae; CH: Subfamily Cactoideae, Tribe Hylocereeae; CN: Subfamily Cactoideae, Tribe Notocacteae; CP: Subfamily Cactoideae, Tribe Pachycereeae; CR: Subfamily Cactoideae, Tribe Rhipsalideae; CT: Subfamily Cactoideae, Tribe Trichocereeae; OA: Subfamily Opuntioideae, Tribe Austrocylindropuntieae; OC: Subfamily Opuntioideae, Tribe Cylindropuntieae; OO: Subfamily Opuntioideae, Tribe Opuntieae; OP: Subfamily Opuntioideae, Tribe Pterocacteae; OT: Subfamily Opuntioideae, Tribe Tephrocacteae; M: Subfamily Maihuenioideae. PE: Subfamily Pereskioideae. The bars in the left graph indicate the percentage of non-invasive species (black) against percentage of invasive species (red) per tribe or subfamily.

Our phylogeny, representing 103 taxa (genera), retrieved three main clades that differed substantially in the proportion of invasive taxa (Fig. 4). Clade 1 included mainly genera of the tribes of the subfamily Cactoideae (with the exception of the tribe Cacteae), and some invasive taxa. Clade 2 comprised genera within the tribe Cacteae (with the exception of *Maihueniopsis* from the tribe Tephrocacteae) and does not include any invasive taxa. Clade 3 (which includes the subfamily Opuntioideae) contains most of the invasive species. Using proportions of invasive taxa/genus, Blomberg's *K* indicated no significant phylogenetic signal of invasiveness (*K* = 0.260; *P* = 0.187), whereas Pagel's *λ* indicated a significant phylogenetic signal for invasiveness (*λ* = 0.991; *P* < 0.01). This phylogenetic signal was not related to human usage, i.e. ornamental trade.

**Figure 5.**
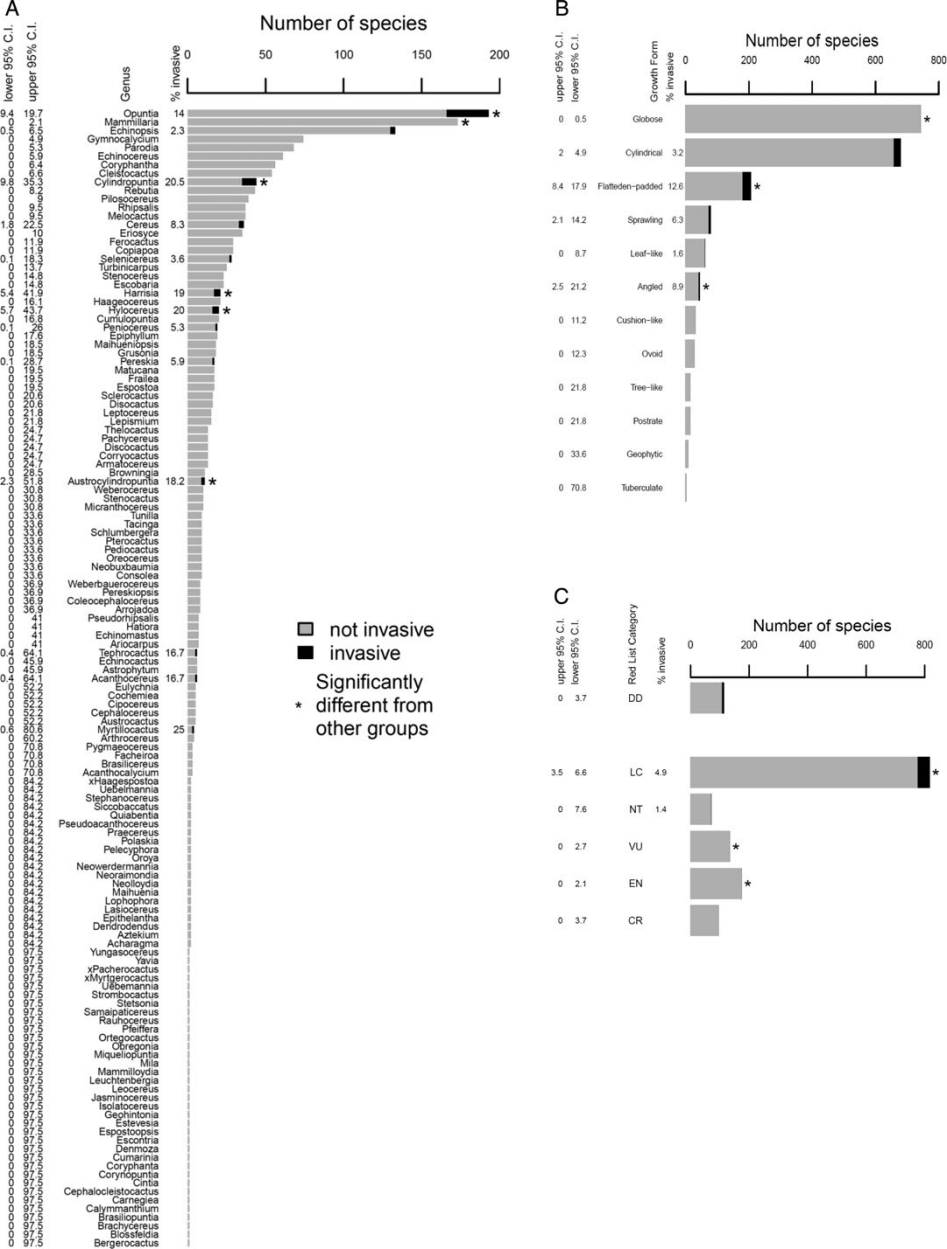
The distribution of invasive cacti within (A) genera, (B) growth forms and (C) IUCN Red List categories. Significance levels were determined by comparing the number of invasive vs. number of non-invasive for any group to all other taxa using Fisher's exact test. Confidence intervals were determined for the per cent invasive or introduced based on an assumption of binomial errors.

At the genus level, the 57 invasive species belong to just 13 of the 130 genera (Fig. 5A). *Opuntia*, *Cylindropuntia*, *Harrisia*, *Hylocereus* and *Austrocylindropuntia* have a significantly higher proportion of invasive species than other genera, while only *Mammillaria* has a significantly lower incidence of invasiveness (it contains no invasive species).

In terms of the 12 main growth forms we considered (Fig. 1), all of the invasive species were cylindrical, flattened-padded, sprawling, leaf-like or angled. The flattened-padded and angled growth forms stand out as having a significantly higher percentage of invasive species than the other growth forms, while the globose growth form is significantly underrepresented in terms of invasiveness (there are no globose invasive taxa) (Fig. 5B).

The latitudinal ranges of invasive *Opuntia* species (21°, *n* = 15) are significantly greater than those of non-invasive species (8.7°, *n* = 42) (*t*-test, *P* < 0.009). As of 2013, the IUCN had assessed 1409 cactus species. All invasive taxa are in the Least Concern, Near Threatened or Data Deficient categories and none of the known invasive species are among the 303 taxa listed in the categories Critically Endangered (CR), Endangered (EN) or Vulnerable (VU) (i.e. species that likely have smaller ranges, Fig. 5C). This provides preliminary evidence for a relationship between native range size and invasiveness.

## Discussion

The results of this study allowed us to draw generalizations that are useful for managing future introductions and invasions. Despite the extensive dissemination of cactus species around the world, only a small proportion of the family is currently known to be invasive. However, these invasive taxa have very large potential ranges globally, including in areas where no cactus invasions have yet been recorded. Cacti are introduced to new areas mainly for ornamentation, but the selection of ornamental species is not based on attributes that favour invasiveness. Invasive taxa are overrepresented in several genera, phylogenetic clades and growth forms. Species that are of conservation concern in their native ranges have not become invasive.

### Proportion of invasive taxa in the family Cactaceae

The main centres of cactus diversity are north-eastern Mexico, the eastern Andes of Bolivia and Argentina and south-eastern Brazil (Mutke and Barthlott 2005). However, species are distributed throughout a large variety of habitats, including hot deserts, sandy coastal stretches, scrublands, dry deciduous forests, high alpine steppes and even tropical rain forests (Barthlott and Hunt 1993). Therefore, there are cactus species that are climatically suited to almost all habitats on Earth. However, only 3 % of the species in the family are currently clearly invasive. It is difficult to say whether this reflects the real extent of invasions or whether the pattern is affected by different levels of reporting and the availability of accurate data, moreover, not all cactus species have been afforded the same opportunities to become invasive. Nonetheless, this pattern has also been observed in other model groups. For example, only between 0.5 and 0.7 % of the global pool of tree and shrub species are currently clearly invasive outside their natural range (Richardson and Rejmánek 2011).

### Potential for further invasions

Currently, most cactus invasions are recorded in Australia, South Africa and Spain. Unsurprisingly, the results of the broad-scale climatic matching identified these three countries as being bioclimatically equivalent to areas within the range of a large number of cactus species. A strong climatic match between native and recipient ranges is recognized as a fundamental requirement for the success of introduced plants (Richardson *et al.* 2011; Richardson and Pyšek 2012). However, many other areas of the world where these species are currently absent are also highlighted as potentially suitable for invasion. This pattern probably reflects differences in introduction effort, and suggests a substantial invasion debt (*sensu* Essl *et al.* 2011) in agreement with the findings for other model groups (e.g. the genera *Casuarina* and *Prosopis*; Potgieter *et al.* 2013; Shackleton *et al.* 2014). Clearly, the natural experiment of plantings of cacti outside their natural ranges is far from complete.

Because efforts directed at prevention of new introductions are the most cost-efficient component of invasive species control strategies (Leung *et al.* 2002), our results emphasize the importance of controlling the introduction of cacti recorded as invasive in Australia, South Africa and Spain to other areas suitable for invasion.

### Reasons for introduction and dissemination

Among the many uses of cacti, the main reason for introductions of species to regions outside their native ranges is the horticulture trade. Cultivation of ornamental cactus species is very popular in temperate regions. There are more than 20 cactus and succulent journals and hundreds of societies around the world, as well as hundreds of cactus and succulent Facebook pages and groups. Moreover, global introductions of new species are likely to occur: just 23 % of the species considered to have ornamental value in the native range have been introduced to other regions.

The use of alien plants for ornamentation is an important driver of introductions and dissemination in many plant groups, and several attributes associated with attractiveness (and hence the popularity of the species for horticulture) are also important for invasiveness. For example, trees used for ornamentation are often selected for their long-lasting displays of brightly coloured fleshy fruits that are attractive to a wide range of generalist seed dispersers (Richardson and Rejmánek 2011). As another example, Australian *Acacia* species used for ornamentation have rapid growth rates and can survive and flourish in nutrient-poor, arid or degraded sites (Richardson *et al.* 2011; Donaldson *et al.* 2014). Ornamental cacti, on the other hand, appear to be selected for features other than those that directly enhance invasiveness; in particular, species that survive without much input and grow slowly are favoured (i.e. more *K*-selected than *r*-selected). The most popular cactus species in the global ornamental trade belong to the genus *Mammillaria* (Novoa *et al.*, unpubl. data); these species are valued for their globose growth form more than any other feature. As no *Mammillaria* spp. are invasive and no globose taxa are invasive (Fig. 5A and B), it is likely that this genus/growth form poses little risk of invasion or impact due to its ecological strategy.

### Correlates of invasiveness

Besides the past and current efforts directed at preventing new introductions of species already known as invasive elsewhere, additional protocols for regulating risk are needed. This is because most contemporary introductions and dissemination of cacti are of ornamental taxa, many of which do not have well-documented introduction/invasion histories. Our results suggest that delimitations based on membership to genera, position in the phylogeny, growth form and native range size need to be considered to produce objective and defendable approaches for formal risk assessments.

Primary attention with regard to invasiveness in cacti needs to be given to taxa in the 13 genera of Cactaceae that consistently display invasive tendencies. These genera (comprising 538 species) share certain characteristics which include prolific fruiting, strong vegetative reproduction and effective dispersal mechanisms (Walters *et al.* 2011). This pattern is particularly seen in the ‘opuntoid cacti’ (i.e. the genera *Austrocylindropuntia*, *Cylindropuntia* and *Opuntia*), which have been classed together as Weeds of National Significance in Australia (Lloyd and Reeves 2014). Our phylogenetic analysis (Fig. 4) provides support for this approach. Invasive taxa are relatively common in the Opuntioideae clade, whereas the incidence of invasiveness in the tribe Cacteae is zero. Interestingly, genera from these clades are well represented in the global horticultural trade, and presumably have similar levels of dissemination and introduction effort (Novoa *et al*., unpubl. data). This pattern in Cactaceae is similar to that seen in conifers. Twenty-eight of the known invasive conifer taxa belong to one family (Pinaceae) and 21 of these are in a single genus—*Pinus* (Richardson and Rejmánek 2004).

One noticeable feature of Cactaceae is the range of growth forms within the family. Unlike most plant groups studied to date, invasiveness in cacti is strongly associated with particular growth forms. All invasive cacti are angled, cylindrical, flattened-padded or sprawling. The reason for high levels of invasiveness in these growth forms probably relates to the strong ability of taxa in these groups to grow vegetatively from cuttings which can allow for rapid dispersal (Anderson 2001).

It would seem that the same traits that allow some cactus species to become widespread in their native ranges contribute to their ability to overcome abiotic filters and successfully establish in new regions. No cactus species that are of conservation concern in their native ranges have been recorded as invasive, and there is a strong correlation between invasiveness and native range size in, for example, the genus *Opuntia*. A similar pattern has been observed for other model groups. For example, Australian *Acacia* species with large native ranges and low percolation exponents (i.e. high population increase rate) are most likely to be introduced and become naturalized (Hui *et al.* 2011). Large native range size has been shown to be a good predictor of invasiveness and invasion success in many, but not all, plant groups (e.g. Procheş *et al.* 2012; Moodley *et al.* 2013; Potgieter *et al.* 2013).

## Conclusions

Cacti are already among the most widespread and damaging of invasive alien plants in some parts of the world. The huge and growing interest in many cacti for ornamentation has created an important new pathway for the introduction and dissemination of a growing number of cactus taxa around the world. Many new invasion events are expected in the future. There is clearly a need to regulate the movement of cacti recorded as invasive elsewhere (currently only 3 % of the species in the family) to areas suitable for invasion, as well as taxa that pose a high risk of becoming invasive. Results from this study suggest that risk assessment protocols for cacti should evaluate taxa according to genera, position in the phylogeny of the family, growth form, and, potentially, native range size.

## Sources of Funding

Funding for this work was provided by the Working for Water (WfW) Programme of the South African Department of Environmental Affairs and the DST-NRF Centre of Excellence for Invasion Biology (C·I·B) as part of the C·I·B/WfW collaborative research programme on ‘Research for Integrated Management of Invasive Alien Species’. D.M.R. acknowledges additional support from the National Research Foundation (grant 85417) and the Oppenheimer Memorial Trust.

## Contributions by the Authors

A.N., J.R.U.W. and D.M.R. conceived the idea. A.N. collected the data. A.N. and J.R.U.W. ran the statistics, J.J.L.R. built the phylogeny and M.P.R. undertook climate matching. A.N. led the writing with assistance from others.

## Conflicts of Interest Statement

None declared.

## Supporting Information

The following Supporting Information is available in the online version of this article –

**Table S1.** Examples of sources of information on cactus species.

**Table S2.** List of cactus species. *57 species recorded as invasive outside their native range.

**Figure S3.** Potential species richness based on available native records only of 39 invasive cactus species.

**Table S4.** Examples of cacti and succulents Journals.

**Table S5.** The date of foundation of each society is shown.

Additional Information
