# Supplementary material for: Introduced and invasive cactus species: a global review
Source: AoB Plants. 2014 Dec 3;7:plu078. doi: 10.1093/aobpla/plu078 (PMC4318432; doi:10.1093/aobpla/plu078)
Supplement: Additional Information [file supp_7_plu078_index.html]

Introduced and invasive cactus species: a global review — Additional Information 

# Introduced and invasive cactus species: a global review

## Additional Information

Additional Information

**Files in this Data Supplement:**

- Supporting information File 1 - docx file
- Supporting information File 2 - docx file
- Supporting information File 3 - pptx file
- Supporting information File 4 - docx file
- Supporting information File 5 - docx file
