# Supplementary material for: Introduced and invasive cactus species: a global review
Source: AoB Plants. 2014 Dec 3;7:plu078. doi: 10.1093/aobpla/plu078 (PMC4318432; doi:10.1093/aobpla/plu078)
Supplement: Additional Information [file supp_plu078_plu078supp_file2.docx]

Supporting information. File 2. List of Cactus species. *: 57species recorded as invasive outside their native range

*Acanthocalycium ferrarii*

*Acanthocalycium klimpelianum*

*Acanthocalycium spiniflorum*

*Acanthocereus baxaniensis*

*Acanthocereus colombianus*

*Acanthocereus horridus*

*Acanthocereus occidentalis*

*Acanthocereus subinermis*

*Acanthocereus tetragonus**

*Acharagma aguirreana*

*Acharagma roseana*

*Ariocarpus agavoides*

*Ariocarpus bravoanus*

*Ariocarpus fissuratus*

*Ariocarpus kotchebeyanus*

*Ariocarpus retusus*

*Ariocarpus scaphirostris*

*Ariocarpus trigonus*

*Armatocereus arduus*

*Armatocereus brevispinus*

*Armatocereus cartwrightianus*

*Armatocereus godingianus*

*Armatocereus humilis*

*Armatocereus laetus*

*Armatocereus mataranus*

*Armatocereus matucanensis*

*Armatocereus oligogonus*

*Armatocereus procerus*

*Armatocereus rauhii*

*Armatocereus riomajensis*

*Armatocereus rupicola*

*Arrojadoa albiflora*

*Arrojadoa bahiensis*

*Arrojadoa beateae*

*Arrojadoa dinae*

*Arrojadoa eriocaulis*

*Arrojadoa multiflora*

*Arrojadoa penicillata*

*Arrojadoa rhodantha*

*Arthrocereus glaziovii*

*Arthrocereus melanurus*

*Arthrocereus rondonianus*

*Arthrocereus spinosissimus*

*Astrophytum asterias*

*Astrophytum capricorne*

*Astrophytum caput-medusae*

*Astrophytum coahuilense*

*Astrophytum myriostigma*

*Astrophytum ornatum*

*Austrocactus bertinii*

*Austrocactus coxii*

*Austrocactus patagonicus*

*Austrocactus philippii*

*Austrocactus spiniflorus*

*Austrocylindropuntia cylindrica**

*Austrocylindropuntia floccosa*

*Austrocylindropuntia hirschii*

*Austrocylindropuntia lagopus*

*Austrocylindropuntia pachypus*

*Austrocylindropuntia punta-caillan*

*Austrocylindropuntia shaferi*

*Austrocylindropuntia subulata**

*Austrocylindropuntia verschaffeltii*

*Austrocylindropuntia vestita*

*Austrocylindropuntia yanganucensis*

*Aztekium hintonii*

*Aztekium ritteri*

*Bergerocactus emoryi*

*Blossfeldia liliputana*

*Brachycereus nesioticus*

*Brasilicereus estevesii*

*Brasilicereus markgrafii*

*Brasilicereus phaeacanthus*

*Brasiliopuntia brasiliensis*

*Browningia albiceps*

*Browningia altissima*

*Browningia amstutziae*

*Browningia caineana*

*Browningia candelaris*

*Browningia chlorocarpa*

*Browningia columnaris*

*Browningia hertlingiana*

*Browningia microsperma*

*Browningia pilleifera*

*Browningia viridis*

*Calymmanthium substerile*

*Carnegiea gigantea*

*Cephalocereus apicephalium*

*Cephalocereus columna-trajani*

*Cephalocereus nizandensis*

*Cephalocereus senilis*

*Cephalocereus totolapensis*

*Cephalocleistocactus chrysocephalus*

*Cereus adelmarii*

*Cereus aethiops*

*Cereus albicaulis*

*Cereus argentinensis*

*Cereus bicolor*

*Cereus braunii*

*Cereus cochabambensis*

*Cereus comarapanus*

*Cereus estevesii*

*Cereus fernambucensis*

*Cereus fricii*

*Cereus haageanus*

*Cereus hankeanus*

*Cereus hexagonus**

*Cereus hildmannianus**

*Cereus horrispinus*

*Cereus huilunchu*

*Cereus insularis*

*Cereus jamacaru**

*Cereus kroenleinii*

*Cereus lamprospermus*

*Cereus lanosus*

*Cereus mirabella*

*Cereus mortensenii*

*Cereus pachyrhizus*

*Cereus phatnospermus*

*Cereus pierre-braunianus*

*Cereus repandus*

*Cereus roseiflorus*

*Cereus saddianus*

*Cereus spegazzinii*

*Cereus stenogonus*

*Cereus tacuaralensis*

*Cereus trigonodendron*

*Cereus validus*

*Cereus vargasianus*

*Cintia knizei*

*Cipocereus bradei*

*Cipocereus crassisepalus*

*Cipocereus laniflorus*

*Cipocereus minensis*

*Cipocereus pusilliflorus*

*Cleistocactus acanthurus*

*Cleistocactus baumannii*

*Cleistocactus brookeae*

*Cleistocactus buchtienii*

*Cleistocactus candelilla*

*Cleistocactus chotaensis*

*Cleistocactus clavispinus*

*Cleistocactus x crassiserpens*

*Cleistocactus dependens*

*Cleistocactus ferrarii*

*Cleistocactus fieldianus*

*Cleistocactus grossei*

*Cleistocactus hildegardiae*

*Cleistocactus hoffmannii*

*Cleistocactus hyalacanthus*

*Cleistocactus hystrix*

*Cleistocactus icosagonus*

*Cleistocactus jajoanus*

*Cleistocactus laniceps*

*Cleistocactus longiserpens*

*Cleistocactus luribayensis*

*Cleistocactus micropetalus*

*Cleistocactus morawetzianus*

*Cleistocactus muyurinensis*

*Cleistocactus neoroezlii*

*Cleistocactus orthogonus*

*Cleistocactus pachycladus*

*Cleistocactus palhuayensis*

*Cleistocactus paraguariensis*

*Cleistocactus parapetiensis*

*Cleistocactus parviflorus*

*Cleistocactus peculiaris*

*Cleistocactus piraymirensis*

*Cleistocactus plagiostoma*

*Cleistocactus pungens*

*Cleistocactus reae*

*Cleistocactus ritteri*

*Cleistocactus roezlii*

*Cleistocactus samaipatanus*

*Cleistocactus sepium*

*Cleistocactus serpens*

*Cleistocactus sextonianus*

*Cleistocactus smaragdiflorus*

*Cleistocactus strausii*

*Cleistocactus sulcifer*

*Cleistocactus tarijensis*

*Cleistocactus tenuiserpens*

*Cleistocactus tominensis*

*Cleistocactus tupizensis*

*Cleistocactus varispinus*

*Cleistocactus viridiflorus*

*Cleistocactus vulpis-cauda*

*Cleistocactus winteri*

*Cleistocactus xylorhizus*

*Cochemiea halei*

*Cochemiea maritima*

*Cochemiea pondii*

*Cochemiea poselgeri*

*Cochemiea setispina*

*Coleocephalocereus aureus*

*Coleocephalocereus braunii*

*Coleocephalocereus buxbaumianus*

*Coleocephalocereus fluminensis*

*Coleocephalocereus goebelianus*

*Coleocephalocereus pluricostatus*

*Coleocephalocereus purpureus*

*Coleocephalocereus uebelmanniorum*

*Consolea corallicola*

*Consolea falcata*

*Consolea macracantha*

*Consolea millspaughii*

*Consolea moniliformis*

*Consolea nashii*

*Consolea picardae*

*Consolea rubescens*

*Consolea spinosissima*

*Copiapoa ahremephiana*

*Copiapoa angustiflora*

*Copiapoa atacamensis*

*Copiapoa bridgesii*

*Copiapoa calderana*

*Copiapoa cinerascens*

*Copiapoa cinerea*

*Copiapoa conglomerata*

*Copiapoa coquimbana*

*Copiapoa decorticans*

*Copiapoa echinoides*

*Copiapoa esmeraldana*

*Copiapoa fieldleriana*

*Copiapoa grandiflora*

*Copiapoa haseltoniana*

*Copiapoa humilis*

*Copiapoa hypogaea*

*Copiapoa krainziana*

*Copiapoa laui*

*Copiapoa longistaminea*

*Copiapoa malletiana*

*Copiapoa marginata*

*Copiapoa megarhiza*

*Copiapoa mollicula*

*Copiapoa serpentisulcata*

*Copiapoa solaris*

*Copiapoa taltalensis*

*Copiapoa tenuissima*

*Copiapoa tocopillana*

*Corryocactus apiciflorus*

*Corryocactus aureus*

*Corryocactus ayacuchoensis*

*Corryocactus brachypetalus*

*Corryocactus brevistylus*

*Corryocactus chachapoyensis*

*Corryocactus charazanensis*

*Corryocactus erectus*

*Corryocactus huincoensis*

*Corryocactus melanotrichus*

*Corryocactus pulquiensis*

*Corryocactus quadrangularis*

*Corryocactus squarrosus*

*Corynopuntia aggeria*

*Coryphanta compacta*

*Coryphantha calipensis*

*Coryphantha calochlora*

*Coryphantha clavata*

*Coryphantha cornifera*

*Coryphantha cornuta*

*Coryphantha delaetiana*

*Coryphantha difficilis*

*Coryphantha duragensis*

*Coryphantha echinoidea*

*Coryphantha echinus*

*Coryphantha elephantidens*

*Coryphantha erecta*

*Coryphantha georgii*

*Coryphantha glanduligera*

*Coryphantha glassii*

*Coryphantha gracilis*

*Coryphantha grata*

*Coryphantha guerkeana*

*Coryphantha hintoniorum*

*Coryphantha indensis*

*Coryphantha jalpanensis*

*Coryphantha kracikii*

*Coryphantha longicornis*

*Coryphantha macromeris*

*Coryphantha maiz-tablasensis*

*Coryphantha maliterrarum*

*Coryphantha melleospina*

*Coryphantha neglecta*

*Coryphantha nickelsiae*

*Coryphantha octacantha*

*Coryphantha odorata*

*Coryphantha ottonis*

*Coryphantha pallida*

*Coryphantha poselgeriana*

*Coryphantha potosiana*

*Coryphantha pseudoechinus*

*Coryphantha pseudonickelsiae*

*Coryphantha pseudoradians*

*Coryphantha pulleineana*

*Coryphantha pusilliflora*

*Coryphantha pycnacantha*

*Coryphantha radians*

*Coryphantha ramillosa*

*Coryphantha recurvata*

*Coryphantha reduncispina*

*Coryphantha retusa*

*Coryphantha robustispina*

*Coryphantha salinensis*

*Coryphantha sulcata*

*Coryphantha sulcolanata*

*Coryphantha tripugionacantha*

*Coryphantha unicornis*

*Coryphantha vaupeliana*

*Coryphantha vogtherriana*

*Coryphantha werdermannii*

*Coryphantha wohlschlageri*

*Cumarinia odorata*

*Cumulopuntia boliviana*

*Cumulopuntia chichensis*

*Cumulopuntia corotilla*

*Cumulopuntia crassicylindrica*

*Cumulopuntia dactylifera*

*Cumulopuntia frigida*

*Cumulopuntia fulvicoma*

*Cumulopuntia galerasensis*

*Cumulopuntia hystrix*

*Cumulopuntia ignescens*

*Cumulopuntia mistiensis*

*Cumulopuntia pentlandii*

*Cumulopuntia pyrrhacantha*

*Cumulopuntia rossiana*

*Cumulopuntia sphaerica*

*Cumulopuntia ticnamarensis*

*Cumulopuntia tortispina*

*Cumulopuntia tumida*

*Cumulopuntia unguispina*

*Cumulopuntia zehnderi*

*Cylindropuntia abyssi*

*Cylindropuntia acanthocarpa*

*Cylindropuntia alcahes**

*Cylindropuntia anteojoensis*

*Cylindropuntia arbuscula*

*Cylindropuntia bigelovii*

*Cylindropuntia californica*

*Cylindropuntia calmalliana*

*Cylindropuntia caribaea*

*Cylindropuntia cedrosensis*

*Cylindropuntia cholla*

*Cylindropuntia davisii*

*Cylindropuntia echinocarpa*

*Cylindropuntia fulgida**

*Cylindropuntia ganderi*

*Cylindropuntia hystrix*

*Cylindropuntia imbricata**

*Cylindropuntia kleiniae**

*Cylindropuntia leptocaulis**

*Cylindropuntia lindsayi*

*Cylindropuntia molesta*

*Cylindropuntia multigeniculata*

*Cylindropuntia munzii*

*Cylindropuntia pallida*

*Cylindropuntia prolifera*

*Cylindropuntia ramosissima*

*Cylindropuntia rosea**

*Cylindropuntia sanfelipensis*

*Cylindropuntia santamaria*

*Cylindropuntia spinosior**

*Cylindropuntia tesajo*

*Cylindropuntia thurberi*

*Cylindropuntia tunicata**

*Cylindropuntia versicolor*

*Cylindropuntia whipplei*

*Cylindropuntia wolfii*

*Cylindropuntia x congesta*

*Cylindropuntia x deserta*

*Cylindropuntia x fosbergii*

*Cylindropuntia x kelvinensis*

*Cylindropuntia x neoarbuscula*

*Cylindropuntia x tetracantha*

*Cylindropuntia x viridiflora*

*Cylindropuntia x vivipara*

*Dendrodendus nudiflorus*

*Dendrodendus undulosus*

*Denmoza rhodacantha*

*Discocactus bahiensis*

*Discocactus cangaensis*

*Discocactus catingicola*

*Discocactus cephaliaciculosus*

*Discocactus diersianus*

*Discocactus ferricola*

*Discocactus hartmannii*

*Discocactus heptacanthus*

*Discocactus horstii*

*Discocactus placentiformis*

*Discocactus pseudoinsignis*

*Discocactus subterraneo-proliferans*

*Discocactus zehntneri*

*Disocactus ackermannii*

*Disocactus amazonicus*

*Disocactus aurantiacus*

*Disocactus biformis*

*Disocactus cinnabarinus*

*Disocactus eichlamii*

*Disocactus flagelliformis*

*Disocactus kimnachii*

*Disocactus macdougallii*

*Disocactus macranthus*

*Disocactus martianus*

*Disocactus nelsonii*

*Disocactus phyllanthoides*

*Disocactus quezaltecus*

*Disocactus schrankii*

*Disocactus speciosus*

*Echinocactus grusonii*

*Echinocactus horizonthalonius*

*Echinocactus parryi*

*Echinocactus platyacanthus*

*Echinocactus polycephalus*

*Echinocactus texensis*

*Echinocereus adustus*

*Echinocereus apachensis*

*Echinocereus barthelowanus*

*Echinocereus berlandieri*

*Echinocereus bonkerae*

*Echinocereus boyce-thompsonii*

*Echinocereus brandegeei*

*Echinocereus bristolii*

*Echinocereus chisoensis*

*Echinocereus cinerascens*

*Echinocereus coccineus*

*Echinocereus dasyacanthus*

*Echinocereus engelmannii*

*Echinocereus enneacanthus*

*Echinocereus fasciculatus*

*Echinocereus fendleri*

*Echinocereus ferreirianus*

*Echinocereus freudenbergeri*

*Echinocereus grandis*

*Echinocereus klapperi*

*Echinocereus knippelianus*

*Echinocereus laui*

*Echinocereus ledingii*

*Echinocereus leucanthus*

*Echinocereus longisetus*

*Echinocereus mapimiensis*

*Echinocereus maritimus*

*Echinocereus mojavensis*

*Echinocereus nicholii*

*Echinocereus nivosus*

*Echinocereus ortegae*

*Echinocereus palmeri*

*Echinocereus pamanesiorum*

*Echinocereus papillosus*

*Echinocereus parkeri*

*Echinocereus pectinatus*

*Echinocereus pensilis*

*Echinocereus pentalophus*

*Echinocereus polyacanthus*

*Echinocereus poselgeri*

*Echinocereus primolanatus*

*Echinocereus pseudopectinatus*

*Echinocereus pulchellus*

*Echinocereus rayonesensis*

*Echinocereus reichenbachii*

*Echinocereus rigidissimus*

*Echinocereus russanthus*

*Echinocereus scheeri*

*Echinocereus schereri*

*Echinocereus schmollii*

*Echinocereus sciurus*

*Echinocereus scopulorum*

*Echinocereus spinigemmatus*

*Echinocereus stoloniferus*

*Echinocereus stramineus*

*Echinocereus subinermis*

*Echinocereus triglochidiatus*

*Echinocereus viereckii*

*Echinocereus viridiflorus*

*Echinocereus websterianus*

*Echinocereus x roetteri*

*Echinomastus erectocentrus*

*Echinomastus gautii*

*Echinomastus intertextus*

*Echinomastus johnsonii*

*Echinomastus mariposensis*

*Echinomastus unguispinus*

*Echinomastus warnockii*

*Echinopsis adolfofriedrichii*

*Echinopsis ancistrophora*

*Echinopsis angelesii*

*Echinopsis antezanae*

*Echinopsis arboricola*

*Echinopsis arebaloi*

*Echinopsis atacamensis*

*Echinopsis aurea*

*Echinopsis backebergii*

*Echinopsis baldiana*

*Echinopsis bertramiana*

*Echinopsis bolligeriana*

*Echinopsis boyuibensis*

*Echinopsis brasiliensis*

*Echinopsis breviflora*

*Echinopsis bridgesi*

*Echinopsis bruchii*

*Echinopsis x cabrerae*

*Echinopsis caineana*

*Echinopsis cajasensis*

*Echinopsis calliantholilacina*

*Echinopsis callichroma*

*Echinopsis calochlora*

*Echinopsis camarguensis*

*Echinopsis candicans*

*Echinopsis cephalomacrostibas*

*Echinopsis cerdana*

*Echinopsis chalaensis*

*Echinopsis chamaecereus**

*Echinopsis chiloensis*

*Echinopsis chrysantha*

*Echinopsis chrysochete*

*Echinopsis cinnabarina*

*Echinopsis clavata*

*Echinopsis cochabambensis*

*Echinopsis comarapana*

*Echinopsis conaconensis*

*Echinopsis coquimbana*

*Echinopsis coronata*

*Echinopsis cotacajesii*

*Echinopsis crassicaulis*

*Echinopsis cuzcoensis*

*Echinopsis densispina*

*Echinopsis derenbergii*

*Echinopsis deserticola*

*Echinopsis escayachensis*

*Echinopsis eyriesii*

*Echinopsis fabrisii*

*Echinopsis famatimensis*

*Echinopsis ferox*

*Echinopsis formosa*

*Echinopsis friedrichii*

*Echinopsis glauca*

*Echinopsis glaucina*

*Echinopsis haematantha*

*Echinopsis hahniana*

*Echinopsis hammerschmidii*

*Echinopsis hertrichiana*

*Echinopsis huascha*

*Echinopsis huotii*

*Echinopsis hystrichoides*

*Echinopsis ibicuatensis*

*Echinopsis jajoana*

*Echinopsis kladiwaiana*

*Echinopsis klingleriana*

*Echinopsis knuthiana*

*Echinopsis korethroides*

*Echinopsis lageniformis*

*Echinopsis lamprochlora*

*Echinopsis lateritia*

*Echinopsis leucantha*

*Echinopsis litoralis*

*Echinopsis macrogona*

*Echinopsis mamillosa*

*Echinopsis marsoneri*

*Echinopsis mataranensis*

*Echinopsis maximiliana*

*Echinopsis meyeri*

*Echinopsis mieckleyi*

*Echinopsis minuana*

*Echinopsis mirabilis*

*Echinopsis molesta*

*Echinopsis nigra*

*Echinopsis obrepanda*

*Echinopsis oxygona*

*Echinopsis pachanoi*

*Echinopsis pampana*

*Echinopsis pentlandii*

*Echinopsis peruviana*

*Echinopsis pojoensis*

*Echinopsis pseudomamillosa*

*Echinopsis pugionacantha*

*Echinopsis quadratiumbonata*

*Echinopsis rhodotricha*

*Echinopsis riviere-de-caraltii*

*Echinopsis rojasii*

*Echinopsis saltensis*

*Echinopsis sanguiniflora*

*Echinopsis santaensis*

*Echinopsis schickendantzii**

*Echinopsis schieliana*

*Echinopsis schoenii*

*Echinopsis schrieteri*

*Echinopsis scopulicola*

*Echinopsis silvestrii*

*Echinopsis skottsbergii*

*Echinopsis smrziana*

*Echinopsis spachiana**

*Echinopsis spinibarbis*

*Echinopsis strigosa*

*Echinopsis subdenudata*

*Echinopsis sucrensis*

*Echinopsis tacaquirensis*

*Echinopsis taratensis*

*Echinopsis tarijensis*

*Echinopsis tarmaensis*

*Echinopsis tegeleriana*

*Echinopsis terscheckii*

*Echinopsis thelegona*

*Echinopsis thelegonoides*

*Echinopsis thionantha*

*Echinopsis tiegeliana*

*Echinopsis trichosa*

*Echinopsis tubiflora*

*Echinopsis tulhuayacensis*

*Echinopsis tunariensis*

*Echinopsis uyupampensis*

*Echinopsis vasquezii*

*Echinopsis vatteri*

*Echinopsis volliana*

*Echinopsis walteri*

*Echinopsis werdermannii*

*Echinopsis yuquina*

*Epiphyllum anguliger*

*Epiphyllum cartagense*

*Epiphyllum caudatum*

*Epiphyllum columbiense*

*Epiphyllum costaricense*

*Epiphyllum crenatum*

*Epiphyllum floribundum*

*Epiphyllum grandilobum*

*Epiphyllum guatemalense*

*Epiphyllum hookeri*

*Epiphyllum laui*

*Epiphyllum lepidocarpum*

*Epiphyllum oxypetalum*

*Epiphyllum phyllanthus*

*Epiphyllum pittieri*

*Epiphyllum pumilum*

*Epiphyllum rubrocoronatum*

*Epiphyllum thomasianum*

*Epiphyllum trimetrale*

*Epithelantha bokei*

*Epithelantha micromeris*

*Eriosyce aericarpa*

*Eriosyce andreaeana*

*Eriosyce aspillagae*

*Eriosyce aurata*

*Eriosyce bulbocalyx*

*Eriosyce chilensis*

*Eriosyce confinis*

*Eriosyce crispa*

*Eriosyce curvispina*

*Eriosyce engleri*

*Eriosyce esmeraldana*

*Eriosyce garaventae*

*Eriosyce heinrichiana*

*Eriosyce islayensis*

*Eriosyce krausii*

*Eriosyce kunzei*

*Eriosyce laui*

*Eriosyce limariensis*

*Eriosyce marksiana*

*Eriosyce napina*

*Eriosyce occulta*

*Eriosyce odieri*

*Eriosyce omasensis*

*Eriosyce recondita*

*Eriosyce rodentiophila*

*Eriosyce senilis*

*Eriosyce sociabilis*

*Eriosyce strausiana*

*Eriosyce subgibbosa*

*Eriosyce taltalensis*

*Eriosyce tenebrica*

*Eriosyce umadeave*

*Eriosyce vertongenii*

*Eriosyce villicumensis*

*Eriosyce villosa*

*Escobaria albicolumnaria*

*Escobaria alversonii*

*Escobaria chihuahuensis*

*Escobaria cubensis*

*Escobaria dasyacantha*

*Escobaria deserti*

*Escobaria duncanii*

*Escobaria emskoetteriana*

*Escobaria guadalupensis*

*Escobaria hesteri*

*Escobaria laredoi*

*Escobaria lloydii*

*Escobaria minima*

*Escobaria missouriensis*

*Escobaria orcuttii*

*Escobaria organensis*

*Escobaria robbinsorum*

*Escobaria sandbergii*

*Escobaria sneedii*

*Escobaria tuberculosa*

*Escobaria villardii*

*Escobaria vivipara*

*Escobaria zilziana*

*Escontria chiotilla*

*Espostoa baumannii*

*Espostoa blossfeldiorum*

*Espostoa calva*

*Espostoa frutescens*

*Espostoa guentheri*

*Espostoa huanucoensis*

*Espostoa hylaea*

*Espostoa lanata*

*Espostoa lanianuligera*

*Espostoa melanostele*

*Espostoa mirabilis*

*Espostoa nana*

*Espostoa ritteri*

*Espostoa ruficeps*

*Espostoa senilis*

*Espostoa superba*

*Espostoa utcubambensis*

*Espostoopsis dybowskii*

*Estevesia alex-bragae*

*Eulychnia acida*

*Eulychnia breviflora*

*Eulychnia castanea*

*Eulychnia iquiquensis*

*Eulychnia ritteri*

*Facheiroa cephaliomelana*

*Facheiroa squamosa*

*Facheiroa ulei*

*Ferocactus alamosanus*

*Ferocactus chrysacanthus*

*Ferocactus cylindraceus*

*Ferocactus diguetii*

*Ferocactus eastwoodiae*

*Ferocactus echidne*

*Ferocactus emoryi*

*Ferocactus flavovirens*

*Ferocactus fordii*

*Ferocactus glaucescens*

*Ferocactus gracilis*

*Ferocactus haematacanthus*

*Ferocactus hamatacanthus*

*Ferocactus herrehae*

*Ferocactus histrix*

*Ferocactus johnstonianus*

*Ferocactus latispinus*

*Ferocactus lindsayi*

*Ferocactus macrodiscus*

*Ferocactus peninsulae*

*Ferocactus pilosus*

*Ferocactus pottsi*

*Ferocactus robustus*

*Ferocactus santa-maria*

*Ferocactus schwarzii*

*Ferocactus tiburonensis*

*Ferocactus townsendianus*

*Ferocactus viridescens*

*Ferocactus wislizeni*

*Frailea buenekeri*

*Frailea buiningiana*

*Frailea castanea*

*Frailea cataphracta*

*Frailea chiquitana*

*Frailea curvispina*

*Frailea friedrichii*

*Frailea gracillima*

*Frailea grahliana*

*Frailea knippeliana*

*Frailea mammifera*

*Frailea perumbilicata*

*Frailea phaeodisca*

*Frailea pseudopulcherrima*

*Frailea pumila*

*Frailea pygmaea*

*Frailea schilinzkyana*

*Geohintonia mexicana*

*Grusonia aggeria*

*Grusonia agglomerata*

*Grusonia bradtiana*

*Grusonia bulbispina*

*Grusonia clavata*

*Grusonia dumetorum*

*Grusonia emoryi*

*Grusonia grahamii*

*Grusonia Invicta*

*Grusonia kunzei*

*Grusonia marenae*

*Grusonia moelleri*

*Grusonia parishii*

*Grusonia pulchella*

*Grusonia reflexispina*

*Grusonia robertsii*

*Grusonia schottii*

*Grusonia vilis*

*Gymnocalycium albiareolatum*

*Gymnocalycium ambatoense*

*Gymnocalycium amerhauseri*

*Gymnocalycium andreae*

*Gymnocalycium angelae*

*Gymnocalycium anisitsii*

*Gymnocalycium baldianum*

*Gymnocalycium bayrianum*

*Gymnocalycium berchtii*

*Gymnocalycium bodenbenderianum*

*Gymnocalycium borthii*

*Gymnocalycium bruchii*

*Gymnocalycium buenekeri*

*Gymnocalycium calochlorum*

*Gymnocalycium capillaense*

*Gymnocalycium carmianthum*

*Gymnocalycium castellanosii*

*Gymnocalycium catamarcense*

*Gymnocalycium chiquitanum*

*Gymnocalycium deeszianum*

*Gymnocalycium delaetii*

*Gymnocalycium denudatum*

*Gymnocalycium erinaceum*

*Gymnocalycium eurypleurum*

*Gymnocalycium eytianum*

*Gymnocalycium gibbosum*

*Gymnocalycium horstii*

*Gymnocalycium hossei*

*Gymnocalycium hybopleurum*

*Gymnocalycium hyptiacanthum*

*Gymnocalycium kieslingii*

*Gymnocalycium kroenleinii*

*Gymnocalycium leeanum*

*Gymnocalycium leptanthum*

*Gymnocalycium mackieanum*

*Gymnocalycium marianae*

*Gymnocalycium marsoneri*

*Gymnocalycium megalothelon*

*Gymnocalycium mesopotamicum*

*Gymnocalycium mihanovichii*

*Gymnocalycium monvillei*

*Gymnocalycium mostii*

*Gymnocalycium mucidum*

*Gymnocalycium netrelianum*

*Gymnocalycium neuhuberi*

*Gymnocalycium obductum*

*Gymnocalycium ochoterenae*

*Gymnocalycium oenanthemum*

*Gymnocalycium paediophilum*

*Gymnocalycium paraguayense*

*Gymnocalycium parvulum*

*Gymnocalycium pflanzii*

*Gymnocalycium platense*

*Gymnocalycium pugionacanthum*

*Gymnocalycium quehlianum*

*Gymnocalycium ragonesei*

*Gymnocalycium rauschii*

*Gymnocalycium riojense*

*Gymnocalycium ritterianum*

*Gymnocalycium robustum*

*Gymnocalycium rosae*

*Gymnocalycium saglionis*

*Gymnocalycium schickendantzii*

*Gymnocalycium schroederianum*

*Gymnocalycium spegazzinii*

*Gymnocalycium stellatum*

*Gymnocalycium stenopleurum*

*Gymnocalycium striglianum*

*Gymnocalycium stuckertii*

*Gymnocalycium taningaense*

*Gymnocalycium terweemeanum*

*Gymnocalycium tillianum*

*Gymnocalycium uebelmannianum*

*Gymnocalycium uruguayense*

*Haageocereus acranthus*

*Haageocereus albispinus*

*Haageocereus australis*

*Haageocereus chalaensis*

*Haageocereus chilensis*

*Haageocereus chryseus*

*Haageocereus decumbens*

*Haageocereus fascilularis*

*Haageocereus icensis*

*Haageocereus icosagonoides*

*Haageocereus lanugispinus*

*Haageocereus pacalaensis*

*Haageocereus platinospinus*

*Haageocereus pluriflorus*

*Haageocereus pseudomelanostele*

*Haageocereus pseudoversicolor*

*Haageocereus subtilispinus*

*Haageocereus tenuis*

*Haageocereus versicolor*

*Haageocereus vulpes*

*Haageocereus zangalensis*

*xHaagespostoa albisetata*

*xHaagespostoa climaxantha*

*Harrisia adscendens*

*Harrisia alboriginum*

*Harrisia balansae**

*Harrisia brookii*

*Harrisia divaricata*

*Harrisia earlei*

*Harrisia eriophora*

*Harrisia fernowii*

*Harrisia fragans*

*Harrisia gracilis*

*Harrisia hurstii*

*Harrisia martini**

*Harrisia nashii*

*Harrisia pomanensis**

*Harrisia portoricensis*

*Harrisia regelii*

*Harrisia simpsonii*

*Harrisia taetra*

*Harrisia taylori*

*Harrisia tetracantha*

*Harrisia tortuosa**

*Hatiora cylindrica*

*Hatiora epiphylloides*

*Hatiora gaertneri*

*Hatiora herminiae*

*Hatiora rosea*

*Hatiora salicornioides*

*Hatiora x graeseri*

*Hylocereus bronxensis*

*Hylocereus calcaratus*

*Hylocereus costaricensis**

*Hylocereus escuintlensis*

*Hylocereus estebanensis*

*Hylocereus extensus*

*Hylocereus guatemalensis*

*Hylocereus lemairei*

*Hylocereus microcladus*

*Hylocereus minutiflorus*

*Hylocereus monacanthus*

*Hylocereus ocamponis*

*Hylocereus peruvianus*

*Hylocereus polyrhizus**

*Hylocereus purpusii*

*Hylocereus scandens*

*Hylocereus stenopterus*

*Hylocereus triangularis**

*Hylocereus trigonus*

*Hylocereus undatus**

*Isolatocereus dumortieri*

*Jasminocereus thouarsii*

*Lasiocereus fulvus*

*Lasiocereus rupicola*

*Leocereus bahiensis*

*Lepismium aculeatum*

*Lepismium bolivianum*

*Lepismium brevispinum*

*Lepismium crenatum*

*Lepismium cruciforme*

*Lepismium houlletianum*

*Lepismium ianthothele*

*Lepismium incachacanum*

*Lepismium lorentzianum*

*Lepismium lumbricoides*

*Lepismium micranthum*

*Lepismium miyagawae*

*Lepismium monacanthum*

*Lepismium paranganiense*

*Lepismium warmingianum*

*Leptocereus arboreus*

*Leptocereus assurgens*

*Leptocereus carinatus*

*Leptocereus ekmanii*

*Leptocereus grantianus*

*Leptocereus leonii*

*Leptocereus maxonii*

*Leptocereus paniculatus*

*Leptocereus prostratus*

*Leptocereus quadricostatus*

*Leptocereus santamarinae*

*Leptocereus scopulophilus*

*Leptocereus sylvestris*

*Leptocereus weingartianus*

*Leptocereus wrightii*

*Leuchtenbergia principis*

*Lophophora diffusa*

*Lophophora williamsii*

*Maihuenia patagonica*

*Maihuenia poeppigii*

*Maihueniopsis archiconoidea*

*Maihueniopsis atacamensis*

*Maihueniopsis bonnieae*

*Maihueniopsis camachoi*

*Maihueniopsis clavarioides*

*Maihueniopsis colorea*

*Maihueniopsis crassispina*

*Maihueniopsis darwinii*

*Maihueniopsis domeykoensis*

*Maihueniopsis glomerata*

*Maihueniopsis grandiflora*

*Maihueniopsis minuta*

*Maihueniopsis nigrispina*

*Maihueniopsis ovata*

*Maihueniopsis rahmeri*

*Maihueniopsis subterranea*

*Maihueniopsis tarapacana*

*Maihueniopsis wagenknechtii*

*Mammillaria albicans*

*Mammillaria albicoma*

*Mammillaria albiflora*

*Mammillaria albilanata*

*Mammillaria amajacensis*

*Mammillaria angelensis*

*Mammillaria anniana*

*Mammillaria armillata*

*Mammillaria aureilanata*

*Mammillaria backebergiana*

*Mammillaria barbata*

*Mammillaria baumii*

*Mammillaria beneckei*

*Mammillaria blossfeldiana*

*Mammillaria bocasana*

*Mammillaria bocensis*

*Mammillaria boelderliana*

*Mammillaria bombycina*

*Mammillaria boolii*

*Mammillaria brachytrichion*

*Mammillaria brandegeei*

*Mammillaria canelensis*

*Mammillaria capensis*

*Mammillaria carmenae*

*Mammillaria carnea*

*Mammillaria carretii*

*Mammillaria cerralboa*

*Mammillaria chinocephala*

*Mammillaria coahuilensis*

*Mammillaria columbiana*

*Mammillaria compressa*

*Mammillaria crinita*

*Mammillaria crucigera*

*Mammillaria decipiens*

*Mammillaria deherdtiana*

*Mammillaria densispina*

*Mammillaria dioica*

*Mammillaria discolor*

*Mammillaria dixanthocentron*

*Mammillaria duoformis*

*Mammillaria ekmanii*

*Mammillaria elongata*

*Mammillaria eriacantha*

*Mammillaria erythrosperma*

*Mammillaria estebanensis*

*Mammillaria evermanniana*

*Mammillaria fittkaui*

*Mammillaria flavicentra*

*Mammillaria formosa*

*Mammillaria fraileana*

*Mammillaria gasseriana*

*Mammillaria geminispina*

*Mammillaria gigantea*

*Mammillaria glassii*

*Mammillaria glochidiata*

*Mammillaria goodridgei*

*Mammillaria grahamii*

*Mammillaria grusonii*

*Mammillaria guelzowiana*

*Mammillaria guerreronis*

*Mammillaria guillauminiana*

*Mammillaria haageana*

*Mammillaria hahniana*

*Mammillaria halbingeri*

*Mammillaria heidiae*

*Mammillaria hernandezii*

*Mammillaria herrerae*

*Mammillaria hertrichiana*

*Mammillaria heyderi*

*Mammillaria huitzilopochtli*

*Mammillaria humboldtii*

*Mammillaria hutchisoniana*

*Mammillaria insularis*

*Mammillaria jaliscana*

*Mammillaria johnstonii*

*Mammillaria karwinskiana*

*Mammillaria klissingiana*

*Mammillaria kraehenbuehlii*

*Mammillaria lasiacantha*

*Mammillaria laui*

*Mammillaria lenta*

*Mammillaria limonensis*

*Mammillaria linaresensis*

*Mammillaria lindsayi*

*Mammillaria lloydii*

*Mammillaria longiflora*

*Mammillaria longimamma*

*Mammillaria luethyi*

*Mammillaria magallanii*

*Mammillaria magnifica*

*Mammillaria magnimamma*

*Mammillaria mainiae*

*Mammillaria mammillaris*

*Mammillaria manana*

*Mammillaria marcosii*

*Mammillaria marksiana*

*Mammillaria mathildae*

*Mammillaria matudae*

*Mammillaria mazatlanensis*

*Mammillaria melaleuca*

*Mammillaria melanocentra*

*Mammillaria mercadensis*

*Mammillaria meyranii*

*Mammillaria microhelia*

*Mammillaria miegiana*

*Mammillaria mieheana*

*Mammillaria moelleriana*

*Mammillaria morganiana*

*Mammillaria muehlenfordtii*

*Mammillaria multidigitata*

*Mammillaria mystax*

*Mammillaria napina*

*Mammillaria neopalmeri*

*Mammillaria nivosa*

*Mammillaria nunezii*

*Mammillaria orcuttii*

*Mammillaria oteroi*

*Mammillaria parkinsonii*

*Mammillaria pectinifera*

*Mammillaria peninsularis*

*Mammillaria pennispinosa*

*Mammillaria perbella*

*Mammillaria perezdelarosae*

*Mammillaria petrophila*

*Mammillaria petterssonii*

*Mammillaria phitauiana*

*Mammillaria picta*

*Mammillaria pilispina*

*Mammillaria plumosa*

*Mammillaria polyedra*

*Mammillaria polythele*

*Mammillaria pottsii*

*Mammillaria prolifera*

*Mammillaria rekoi*

*Mammillaria rhodantha*

*Mammillaria roseoalba*

*Mammillaria saboae*

*Mammillaria sanchez-mejoradae*

*Mammillaria sartorii*

*Mammillaria schiedeana*

*Mammillaria schumannii*

*Mammillaria schwarzii*

*Mammillaria scrippsiana*

*Mammillaria sempervivi*

*Mammillaria senilis*

*Mammillaria sheldonii*

*Mammillaria solisioides*

*Mammillaria sonorensis*

*Mammillaria sphacelata*

*Mammillaria sphaerica*

*Mammillaria spinosissima*

*Mammillaria standleyi*

*Mammillaria stella-de-tacubaya*

*Mammillaria supertexta*

*Mammillaria surculosa*

*Mammillaria tayloriorum*

*Mammillaria tepexicensis*

*Mammillaria tetrancistra*

*Mammillaria theresae*

*Mammillaria thornberi*

*Mammillaria tonalensis*

*Mammillaria uncinata*

*Mammillaria varieaculeata*

*Mammillaria vetula*

*Mammillaria voburnensis*

*Mammillaria wagneriana*

*Mammillaria weingartiana*

*Mammillaria wiesingeri*

*Mammillaria winterae*

*Mammillaria wrightii*

*Mammillaria xaltianguensis*

*Mammillaria zephyranthoides*

*Mammillaria zublerae*

*Mammilloydia candida*

*Matucana aurantiaca*

*Matucana aureiflora*

*Matucana comacephala*

*Matucana formosa*

*Matucana fruticosa*

*Matucana haynei*

*Matucana huagalensis*

*Matucana intertexta*

*Matucana krahnii*

*Matucana madisoniorum*

*Matucana oreodoxa*

*Matucana paucicostata*

*Matucana polzii*

*Matucana pujupatii*

*Matucana ritteri*

*Matucana tuberculata*

*Matucana webwebaueri*

*Melocactus x albicephalus*

*Melocactus andinus*

*Melocactus azureus*

*Melocactus bahiensis*

*Melocactus bellavistensis*

*Melocactus braunii*

*Melocactus broadwayi*

*Melocactus caroli-linnaei*

*Melocactus concinnus*

*Melocactus conoideus*

*Melocactus curvispinus*

*Melocactus deinacanthus*

*Melocactus ernestii*

*Melocactus estevesii*

*Melocactus glaucescens*

*Melocactus harlowii*

*Melocactus x horridus*

*Melocactus intortus*

*Melocactus lanssensianus*

*Melocactus lemairei*

*Melocactus levitestatus*

*Melocactus macracanthos*

*Melocactus matanzanus*

*Melocactus mazelianus*

*Melocactus neryi*

*Melocactus oreas*

*Melocactus pachyacanthus*

*Melocactus paucispinus*

*Melocactus perezassoi*

*Melocactus peruvianus*

*Melocactus praerupticola*

*Melocactus salvadorensis*

*Melocactus schatzlii*

*Melocactus smithii*

*Melocactus stramineus*

*Melocactus violaceus*

*Melocactus zehntneri*

*Micranthocereus albicephalus*

*Micranthocereus auriazurensis*

*Micranthocereus dolichospermaticus*

*Micranthocereus estevesii*

*Micranthocereus flaviflorus*

*Micranthocereus hofackerianus*

*Micranthocereus polyanthus*

*Micranthocereus purpureus*

*Micranthocereus streckeri*

*Micranthocereus violaciflorus*

*Mila caespitosa*

*Miqueliopuntia miquelii*

*xMyrtgerocactus lindsayi*

*Myrtillocactus cochal*

*Myrtillocactus eichlamii*

*Myrtillocactus geometrizans**

*Myrtillocactus schenckii*

*Neobuxbaumia euphorbioides*

*Neobuxbaumia laui*

*Neobuxbaumia macrocephala*

*Neobuxbaumia mezcalaensis*

*Neobuxbaumia multiareolata*

*Neobuxbaumia polylopha*

*Neobuxbaumia scoparia*

*Neobuxbaumia squamulosa*

*Neobuxbaumia tetetzo*

*Neolloydia conoidea*

*Neolloydia matehualensis*

*Neoraimondia arequipensis*

*Neoraimondia herzogiana*

*Neowerdermannia chilensis*

*Neowerdermannia vorwerkii*

*Obregonia denegrii*

*Opuntia abjecta*

*Opuntia acaulis*

*Opuntia aciculata*

*Opuntia alko-tuna*

*Opuntia ammophila**

*Opuntia amyclaea**

*Opuntia anacantha*

*Opuntia antillana*

*Opuntia apurimacensis*

*Opuntia arcei*

*Opuntia armata*

*Opuntia assumptionis*

*Opuntia atrispina*

*Opuntia atropes*

*Opuntia atrovirens*

*Opuntia auberi**

*Opuntia aurantiaca**

*Opuntia arechavaletae*

*Opuntia aurea*

*Opuntia aureispina*

*Opuntia austrina*

*Opuntia azurea*

*Opuntia basilaris*

*Opuntia bella*

*Opuntia bensonii*

*Opuntia bisetosa*

*Opuntia boldinghii*

*Opuntia bonplandii*

*Opuntia borinquensis*

*Opuntia bravoana*

*Opuntia canterae*

*Opuntia caracassana*

*Opuntia cardiosperma*

*Opuntia chaffeyi*

*Opuntia chakensis*

*Opuntia chavena*

*Opuntia chihuahuensis*

*Opuntia chisosensis**

*Opuntia chlorotica*

*Opuntia cochabambensis*

*Opuntia cochenillifera**

*Opuntia cognata*

*Opuntia colubrina*

*Opuntia conjungens*

*Opuntia crassa**

*Opuntia crystalenia*

*Opuntia curassavica*

*Opuntia cymochila*

*Opuntia darrahiana*

*Opuntia deamii*

*Opuntia decumbens*

*Opuntia dejecta**

*Opuntia delaetiana*

*Opuntia depauperata*

*Opuntia depressa*

*Opuntia dillenii**

*Opuntia discolor*

*Opuntia durangensis*

*Opuntia echios*

*Opuntia eichlamii*

*Opuntia ekmanii*

*Opuntia elata**

*Opuntia elatior**

*Opuntia elizondoana*

*Opuntia ellisiana*

*Opuntia engelmannii**

*Opuntia excelsa*

*Opuntia feroacantha*

*Opuntia ficus-indica**

*Opuntia fragilis*

*Opuntia fuliginosa*

*Opuntia galapageia*

*Opuntia gosseliniana*

*Opuntia guatemalensis*

*Opuntia guilanchi*

*Opuntia helleri*

*Opuntia hondurensis*

*Opuntia howeyi*

*Opuntia huajuapensis**

*Opuntia humifusa**

*Opuntia hyptiacantha*

*Opuntia inaequilateralis*

*Opuntia inaperta*

*Opuntia infesta*

*Opuntia insularis*

*Opuntia jaliscana*

*Opuntia jamaicensis*

*Opuntia joconostle*

*Opuntia karwinskiana*

*Opuntia laevis*

*Opuntia lagunae*

*Opuntia larreyi*

*Opuntia lasiacantha*

*Opuntia lata*

*Opuntia leucotricha**

*Opuntia lilae*

*Opuntia limitata*

*Opuntia littoralis*

*Opuntia lutea*

*Opuntia macrocentra*

*Opuntia macrorhiza*

*Opuntia martiniana*

*Opuntia megacantha*

*Opuntia megapotamica*

*Opuntia megarhiza*

*Opuntia megasperma*

*Opuntia microdasys**

*Opuntia mieckleyi*

*Opuntia monocantha**

*Opuntia montevideensis*

*Opuntia nejapensis*

*Opuntia neochrysacantha*

*Opuntia nuda*

*Opuntia orbiculata*

*Opuntia oricola*

*Opuntia pachona*

*Opuntia pachyrrhiza*

*Opuntia pailana*

*Opuntia pampeana*

*Opuntia paraguayensis*

*Opuntia parviclada*

*Opuntia penicilligera*

*Opuntia pennellii*

*Opuntia phaeacantha**

*Opuntia pilifera*

*Opuntia pinkavae*

*Opuntia pittieri*

*Opuntia pituitosa*

*Opuntia polyacantha*

*Opuntia pottsii*

*Opuntia prasina*

*Opuntia puberula*

*Opuntia pubescens**

*Opuntia pumila*

*Opuntia pusilla*

*Opuntia pycnantha*

*Opuntia pyriformis*

*Opuntia pyrrhantha*

*Opuntia quimilo*

*Opuntia quitensis*

*Opuntia rastrera*

*Opuntia repens*

*Opuntia rileyi*

*Opuntia ritteri*

*Opuntia robinsonnii*

*Opuntia roborensis*

*Opuntia robusta**

*Opuntia rufida*

*Opuntia salagria*

*Opuntia salmiana**

*Opuntia salvadorensis*

*Opuntia sanguinea*

*Opuntia santa-rita*

*Opuntia saxicola*

*Opuntia scheeri*

*Opuntia schickendantzii*

*Opuntia schumannii*

*Opuntia securigera*

*Opuntia soederstromiana*

*Opuntia spinulifera**

*Opuntia spraguei*

*Opuntia stenarthra*

*Opuntia stenopetala*

*Opuntia streptacantha**

*Opuntia stricta**

*Opuntia strigil*

*Opuntia subsphaerocarpa*

*Opuntia sulfurea*

*Opuntia tapona*

*Opuntia taylori*

*Opuntia tehuacana*

*Opuntia tehuantepecana*

*Opuntia tenuiflora*

*Opuntia tomentella*

*Opuntia tomentosa**

*Opuntia triacantha*

*Opuntia tuna**

*Opuntia turbinata*

*Opuntia undulata*

*Opuntia urbaniana*

*Opuntia velutina*

*Opuntia viridirubra*

*Opuntia vitelliniflora*

*Opuntia wetmorei*

*Opuntia wilcoxii*

*Opuntia x aequatorialis*

*Opuntia x bakeri*

*Opuntia x columbiana*

*Opuntia x cubensis*

*Opuntia x curvospina*

*Opuntia x lucayana*

*Opuntia x occidentalis*

*Opuntia x spinosibacca*

*Opuntia x vaseyi*

*Opuntia x wootonii*

*Oreocereus celsianus*

*Oreocereus doelzianus*

*Oreocereus hempelianus*

*Oreocereus leucotrichus*

*Oreocereus pseudofossulatus*

*Oreocereus ritteri*

*Oreocereus tacnaensis*

*Oreocereus trollii*

*Oreocereus varicolor*

*Oroya borchersii*

*Oroya peruviana*

*Ortegocactus macdougallii*

*xPacherocactus orcuttii*

*Pachycereus fulviceps*

*Pachycereus gatesii*

*Pachycereus gaumeri*

*Pachycereus grandis*

*Pachycereus hollianus*

*Pachycereus lepidanthus*

*Pachycereus marginatus*

*Pachycereus militaris*

*Pachycereus pecten-aboriginum*

*Pachycereus pringlei*

*Pachycereus schottii*

*Pachycereus tepamo*

*Pachycereus weberi*

*Parodia alacriportana*

*Parodia allosiphon*

*Parodia arnostiana*

*Parodia aureicentra*

*Parodia ayopayana*

*Parodia buiningii*

*Parodia carambeiensis*

*Parodia chrysacanthion*

*Parodia columnaris*

*Parodia comarapana*

*Parodia commutans*

*Parodia concinna*

*Parodia crassigibba*

*Parodia curvispina*

*Parodia erinacea*

*Parodia erubescens*

*Parodia formosa*

*Parodia fusca*

*Parodia gaucha*

*Parodia glaucina*

*Parodia haselbergii*

*Parodia hausteiniana*

*Parodia herteri*

*Parodia horstii*

*Parodia langsdorfii*

*Parodia leninghausii*

*Parodia linkii*

*Parodia maassii*

*Parodia magnifica*

*Parodia mammulosa*

*Parodia meonacantha*

*Parodia microsperma*

*Parodia mueller-melchersii*

*Parodia muricata*

*Parodia neoarechavaletae*

*Parodia neohorstii*

*Parodia nigrispina*

*Parodia nivosa*

*Parodia nothominuscula*

*Parodia nothorauschii*

*Parodia ocampoi*

*Parodia ottonis*

*Parodia oxycostata*

*Parodia penicillata*

*Parodia permutata*

*Parodia procera*

*Parodia rechensis*

*Parodia ritteri*

*Parodia rudibuenekeri*

*Parodia rutilans*

*Parodia saint-pieana*

*Parodia schumanniana*

*Parodia schwebsiana*

*Parodia scopa*

*Parodia sellowii*

*Parodia stockingeri*

*Parodia stuemeri*

*Parodia subterranea*

*Parodia tabularis*

*Parodia taratensis*

*Parodia tenuicylindrica*

*Parodia tilcarensis*

*Parodia tuberculata*

*Parodia turbinata*

*Parodia turecekiana*

*Parodia warasii*

*Parodia werdermanniana*

*Parodia werneri*

*Pediocactus bradyi*

*Pediocactus despainii*

*Pediocactus hermannii*

*Pediocactus knowltonii*

*Pediocactus nigrispinus*

*Pediocactus paradinei*

*Pediocactus peeblesianus*

*Pediocactus simpsonii*

*Pediocactus winkleri*

*Pelecyphora aselliformis*

*Pelecyphora strobiliformis*

*Peniocereus castellae*

*Peniocereus chiapensis*

*Peniocereus cuixmalensis*

*Peniocereus fosterianus*

*Peniocereus greggii*

*Peniocereus hirschtianus*

*Peniocereus johnstonii*

*Peniocereus lazaro-cardenasii*

*Peniocereus macdougallii*

*Peniocereus maculatus*

*Peniocereus marianus*

*Peniocereus oaxacensis*

*Peniocereus occidentalis*

*Peniocereus rosei*

*Peniocereus serpentinus**

*Peniocereus striatus*

*Peniocereus tepalcatepecanus*

*Peniocereus viperinus*

*Peniocereus zopilotensis*

*Pereskia aculeata**

*Pereskia aureiflora*

*Pereskia bahiensis*

*Pereskia bleo*

*Pereskia diaz-romeroana*

*Pereskia grandiflora*

*Pereskia guamacho*

*Pereskia horrida*

*Pereskia lychnidiflora*

*Pereskia marcanoi*

*Pereskia nemorosa*

*Pereskia portulacifolia*

*Pereskia quinqueyana*

*Pereskia sacharosa*

*Pereskia stenantha*

*Pereskia weberiana*

*Pereskia zinniiflora*

*Pereskiopsis aquosa*

*Pereskiopsis blakeana*

*Pereskiopsis diguetii*

*Pereskiopsis gatesii*

*Pereskiopsis kellermanii*

*Pereskiopsis porteri*

*Pereskiopsis rotundifolia*

*Pereskiopsis spathulata*

*Pfeiffera asuntapatensis*

*Pilosocereus albisummus*

*Pilosocereus alensis*

*Pilosocereus arrabidae*

*Pilosocereus aureispinus*

*Pilosocereus aurisetus*

*Pilosocereus azulensis*

*Pilosocereus brasiliensis*

*Pilosocereus catingicola*

*Pilosocereus chrysacanthus*

*Pilosocereus chrysostele*

*Pilosocereus collinsii*

*Pilosocereus densiareolatus*

*Pilosocereus diersianus*

*Pilosocereus estevesii*

*Pilosocereus flavipulvinatus*

*Pilosocereus flexibilispinus*

*Pilosocereus floccosus*

*Pilosocereus frewenii*

*Pilosocereus fulvilanatus*

*Pilosocereus glaucochrous*

*Pilosocereus gounellei*

*Pilosocereus lanuginosus*

*Pilosocereus leucocephalus*

*Pilosocereus machrisii*

*Pilosocereus magnificus*

*Pilosocereus multicostatus*

*Pilosocereus occultiflorus*

*Pilosocereus oligolepis*

*Pilosocereus pachycladus*

*Pilosocereus pentaedrophorus*

*Pilosocereus piauhyensis*

*Pilosocereus polygonus*

*Pilosocereus purpusii*

*Pilosocereus quadricentralis*

*Pilosocereus royenii*

*Pilosocereus x subsimilis*

*Pilosocereus tuberculatus*

*Pilosocereus ulei*

*Pilosocereus vilaboensis*

*Polaskia chende*

*Polaskia chichipe*

*Praecereus euchlorus*

*Praecereus saxicola*

*Pseudoacanthocereus brasiliensis*

*Pseudoacanthocereus sicariguensis*

*Pseudorhipsalis acuminata*

*Pseudorhipsalis alata*

*Pseudorhipsalis amazonica*

*Pseudorhipsalis himantoclada*

*Pseudorhipsalis horichii*

*Pseudorhipsalis lankesteri*

*Pseudorhipsalis ramulosa*

*Pterocactus araucanus*

*Pterocactus australis*

*Pterocactus fischeri*

*Pterocactus gonjianii*

*Pterocactus hickenii*

*Pterocactus megiolii*

*Pterocactus reticulatus*

*Pterocactus tuberosus*

*Pterocactus valentinii*

*Pygmaeocereus bieblii*

*Pygmaeocereus bylesianus*

*Pygmaeocereus familiaris*

*Quiabentia verticillata*

*Quiabentia zehntneri*

*Rauhocereus riosaniensis*

*Rebutia albiflora*

*Rebutia albipectinata*

*Rebutia arenacea*

*Rebutia aureiflora*

*Rebutia brunescens*

*Rebutia caineana*

*Rebutia canigueralii*

*Rebutia cardenasiana*

*Rebutia cintia*

*Rebutia cylindrica*

*Rebutia deminuta*

*Rebutia einsteinii*

*Rebutia fabrisii*

*Rebutia fidaiana*

*Rebutia fiebrigii*

*Rebutia flavistyla*

*Rebutia fulviseta*

*Rebutia gonjianii*

*Rebutia heliosa*

*Rebutia huasiensis*

*Rebutia krugerae*

*Rebutia leucanthema*

*Rebutia marsoneri*

*Rebutia mentosa*

*Rebutia minuscula*

*Rebutia narvaecensis*

*Rebutia neocumingii*

*Rebutia neumanniana*

*Rebutia nigricans*

*Rebutia oligacantha*

*Rebutia padcayensis*

*Rebutia perplexa*

*Rebutia pseudodeminuta*

*Rebutia pulvinosa*

*Rebutia pygmaea*

*Rebutia ritteri*

*Rebutia simoniana*

*Rebutia spegazziniana*

*Rebutia spinosissima*

*Rebutia steinbachii*

*Rebutia steinmannii*

*Rebutia wessneriana*

*Rebutia xanthocarpa*

*Rhipsalis agudoensis*

*Rhipsalis baccifera*

*Rhipsalis burchellii*

*Rhipsalis campos-portoana*

*Rhipsalis cereoides*

*Rhipsalis cereuscula*

*Rhipsalis clavata*

*Rhipsalis crispata*

*Rhipsalis cuneata*

*Rhipsalis dissimilis*

*Rhipsalis elliptica*

*Rhipsalis ewaldiana*

*Rhipsalis floccosa*

*Rhipsalis goebeliana*

*Rhipsalis grandiflora*

*Rhipsalis hoelleri*

*Rhipsalis juengeri*

*Rhipsalis lindbergiana*

*Rhipsalis mesembryanthemoides*

*Rhipsalis micrantha*

*Rhipsalis neves-armondii*

*Rhipsalis oblonga*

*Rhipsalis occidentalis*

*Rhipsalis olivifera*

*Rhipsalis ormindoi*

*Rhipsalis pacheco-leonis*

*Rhipsalis pachyptera*

*Rhipsalis paradoxa*

*Rhipsalis pentaptera*

*Rhipsalis pilocarpa*

*Rhipsalis pulchra*

*Rhipsalis puniceodiscus*

*Rhipsalis russellii*

*Rhipsalis sulcata*

*Rhipsalis teres*

*Rhipsalis triangularis*

*Rhipsalis trigona*

*Samaipaticereus corroanus*

*Schlumbergera kautskyi*

*Schlumbergera microsphaerica*

*Schlumbergera opuntioides*

*Schlumbergera orssichiana*

*Schlumbergera russelliana*

*Schlumbergera truncate*

*Schlumbergera x buckleyi*

*Schlumbergera x exotica*

*Schlumbergera x reginae*

*Sclerocactus brevihamatus*

*Sclerocactus brevispinus*

*Sclerocactus glaucus*

*Sclerocactus mesae-verdae*

*Sclerocactus nyensis*

*Sclerocactus papyracanthus*

*Sclerocactus parviflorus*

*Sclerocactus polyancistrus*

*Sclerocactus pubispinus*

*Sclerocactus scheeri*

*Sclerocactus sileri*

*Sclerocactus spinosior*

*Sclerocactus uncinatus*

*Sclerocactus wetlandicus*

*Sclerocactus whipplei*

*Sclerocactus wrightiae*

*Selenicereus anthonyanus*

*Selenicereus atropilosus*

*Selenicereus boeckmannii*

*Selenicereus brevispinus*

*Selenicereus chontalensis*

*Selenicereus chrysocardium*

*Selenicereus coniflorus*

*Selenicereus donkelaari*

*Selenicereus grandiflorus*

*Selenicereus hamatus*

*Selenicereus hondurensis*

*Selenicereus inermis*

*Selenicereus innesii*

*Selenicereus macdonaldiae**

*Selenicereus megalanthus*

*Selenicereus murrillii*

*Selenicereus nelsonii*

*Selenicereus pteranthus*

*Selenicereus rubineus*

*Selenicereus setaceus*

*Selenicereus spinulosus*

*Selenicereus testudo*

*Selenicereus tricae*

*Selenicereus urbanianus*

*Selenicereus vagans*

*Selenicereus validus*

*Selenicereus wercklei*

*Selenicereus wittii*

*Siccobaccatus estevesii*

*Siccobaccatus insigniflorus*

*Stenocactus coptonogonus*

*Stenocactus crispatus*

*Stenocactus hastatus*

*Stenocactus multicostatus*

*Stenocactus obvallatus*

*Stenocactus ochoterenanus*

*Stenocactus phyllacanthus*

*Stenocactus rectispinus*

*Stenocactus sulfureus*

*Stenocactus vaupelianus*

*Stenocereus alamosensis*

*Stenocereus aragonii*

*Stenocereus beneckei*

*Stenocereus chacalapensis*

*Stenocereus chrysocarpus*

*Stenocereus eichlamii*

*Stenocereus eruca*

*Stenocereus fimbriatus*

*Stenocereus fricii*

*Stenocereus griseus*

*Stenocereus gummosus*

*Stenocereus kerberi*

*Stenocereus laevigatus*

*Stenocereus martinezii*

*Stenocereus montanus*

*Stenocereus pruinosus*

*Stenocereus queretaroensis*

*Stenocereus quevedonis*

*Stenocereus standleyi*

*Stenocereus stellatus*

*Stenocereus thurberi*

*Stenocereus treleasei*

*Stenocereus yunckeri*

*Stephanocereus leucostele*

*Stephanocereus luetzelburgii*

*Stetsonia coryne*

*Strombocactus disciformis*

*Tacinga braunii*

*Tacinga estevesii*

*Tacinga funalis*

*Tacinga inamoena*

*Tacinga palmadora*

*Tacinga saxatilis*

*Tacinga subcylindrica*

*Tacinga werneri*

*Tacinga x quipa*

*Tephrocactus alexanderi*

*Tephrocactus aoracanthus*

*Tephrocactus articulatus**

*Tephrocactus geometricus*

*Tephrocactus molinensis*

*Tephrocactus weberi*

*Thelocactus bicolor*

*Thelocactus buekii*

*Thelocactus conothelos*

*Thelocactus garciae*

*Thelocactus hastifer*

*Thelocactus heterochromus*

*Thelocactus hexaedrophorus*

*Thelocactus lausseri*

*Thelocactus leucacanthus*

*Thelocactus macdowellii*

*Thelocactus rinconensis*

*Thelocactus setispinus*

*Thelocactus tulensis*

*Tunilla albisetacens*

*Tunilla chilensis*

*Tunilla corrugata*

*Tunilla erectoclada*

*Tunilla ianthinantha*

*Tunilla minuscula*

*Tunilla orurensis*

*Tunilla silvestris*

*Tunilla soehrensii*

*Turbinicarpus alonsoi*

*Turbinicarpus beguinii*

*Turbinicarpus bonatzii*

*Turbinicarpus booleanus*

*Turbinicarpus gielsdorfianus*

*Turbinicarpus hoferi*

*Turbinicarpus horripilus*

*Turbinicarpus jauernigii*

*Turbinicarpus knuthianus*

*Turbinicarpus laui*

*Turbinicarpus lophophoroides*

*Turbinicarpus mandragora*

*Turbinicarpus x mombergii*

*Turbinicarpus pailanus*

*Turbinicarpus pseudomacrochele*

*Turbinicarpus pseudopectinatus*

*Turbinicarpus rioverdensis*

*Turbinicarpus saueri*

*Turbinicarpus schmiedickeanus*

*Turbinicarpus subterraneus*

*Turbinicarpus swobodae*

*Turbinicarpus valdezianus*

*Turbinicarpus viereckii*

*Turbinicarpus ysabelae*

*Turbinicarpus zaragosae*

*Uebelmannia gummifera*

*Uebelmannia pectinifera*

*Uebemannia buiningii*

*Weberbauerocereus churinensis*

*Weberbauerocereus cuzcoensis*

*Weberbauerocereus johnsonii*

*Weberbauerocereus longicomus*

*Weberbauerocereus rauhii*

*Weberbauerocereus torataensis*

*Weberbauerocereus weberbaueri*

*Weberbauerocereus winterianus*

*Weberocereus biolleyi*

*Weberocereus bradei*

*Weberocereus frohningiorum*

*Weberocereus glaber*

*Weberocereus imitans*

*Weberocereus panamensis*

*Weberocereus rosei*

*Weberocereus tonduzii*

*Weberocereus trichophorus*

*Weberocereus tunilla*

*Yavia cryptocarpa*

*Yungasocereus inquisiv*
