# Supplementary material for: Introduced and invasive cactus species: a global review
Source: AoB Plants. 2014 Dec 3;7:plu078. doi: 10.1093/aobpla/plu078 (PMC4318432; doi:10.1093/aobpla/plu078)
Supplement: Additional Information [file supp_plu078_plu078supp_file4.docx]

Supporting information. File 4. Examples of cacti and Succulents Journals

| **North America**  The cactus and succulent journal. California.  Saguaroland Bulletin. Phoenix.  Cactaceas y Suculentas Mexicanas. Mexico, D. F. |
| --- |
| **Europe**  Cactus. France.  Cactusvrieden. Belgium.  Succulenta. Netherlands  Stackelpost. Germany.  Sukkulentenkunde. Switzerland.  The Cactus Explorers Club Journal. UK  CACTUS-ADVENTURES. Spain.  Cactus & Co. Italy. |
| **Australia and New Zealand**  The Spine. Victoria.  Cactus and Succulent Journal. New South Wales.  New Zealand Cactus and Succulent Journal. Auckland. |
| **Japan**  Succulentarum Japonica. Tokyo.  Journal of the Cactus and Succulent Society of Japan. Kyoto.  The Study of Cactus. Kanagawaken.  Shaboten-sha. Kanagawa. |
