# Supplementary material for: Introduced and invasive cactus species: a global review
Source: AoB Plants. 2014 Dec 3;7:plu078. doi: 10.1093/aobpla/plu078 (PMC4318432; doi:10.1093/aobpla/plu078)
Supplement: Additional Information [file supp_plu078_plu078supp_file5.docx]

Supporting information. File 5. Examples of cactus and succulent societies. The date of foundation of each society is shown.

| **Australia**  The Cactus and Succulent Society of Australia Inc (CSSA). It is the oldest Cactus and Succulent Society in Australia, established in Melbourne in 1927.  The Cactus and Succulent Society of New South Wales Inc. 1957.  Cactus and Succulent Society of Queensland Inc. 1963.  Cactus and Succulent Society of South Australia. 1964. |
| --- |
| **Canada**  Toronto Cactus and Succulent Club. 1977  Victoria Cactus & Succulent Society. 1992. |
| **Europe**  Kakteenfreunden Berliner. 1893.  Magyar Kaktuszgyűjtők Országos Egyesülete. 1971  Oslo sukkulentforening. 1980.  The Dublin and District Cactus and Succulent Society. 1992  Société Succulentophile Francilienne. 1994.  ASAC, Asociación dels amics dels cactus. 1996  Cactus & Co. International Society. 1996 |
| **India**  The Indian Society of Cactus & Succulents (ISOCS). 1984. |
| **Japan**  Kagawa Cactus Club. 1965. |
| **Mexico**  La Sociedad Mexicana de Cactologia. 1951.  The Cactus and Succulent Society of New Mexico (CSSNM). 1955.  Sociedad de Cactáceas y Suculentas del Estado de Nuevo León. 2001. |
| **New Zealand**  Cactus & Succulent Society of New Zealand Inc. 1947. |
| **Philippines**  Cactus and Succulent Society of the Philippines. 1994. |
| **South Africa**  Succulent Society of South Africa. 1963. |
| **South America and the Caribbaen**  Sociedad Peruana de Cactus y Suculentas (SPECS). 1987.  Sociedad Latinoamericana y del Caribe de Cactáceas y otras Suculentas. 1989. |
| **United States**  The Cactus and Succulent Society of America (CSSA). 1929  The Cactus & Succulent Society of Hawaii. 1965.  The Cactus and Succulent Society of Southern Nevada (CSSSN). 1976.  Cactus and Succulent Society of Massachusetts. 1991. |
