# Supplementary figures and images for: Introduced and invasive cactus species: a global review
Source: AoB Plants. 2014 Dec 3;7:plu078. doi: 10.1093/aobpla/plu078 (PMC4318432; doi:10.1093/aobpla/plu078)

## Slide 1
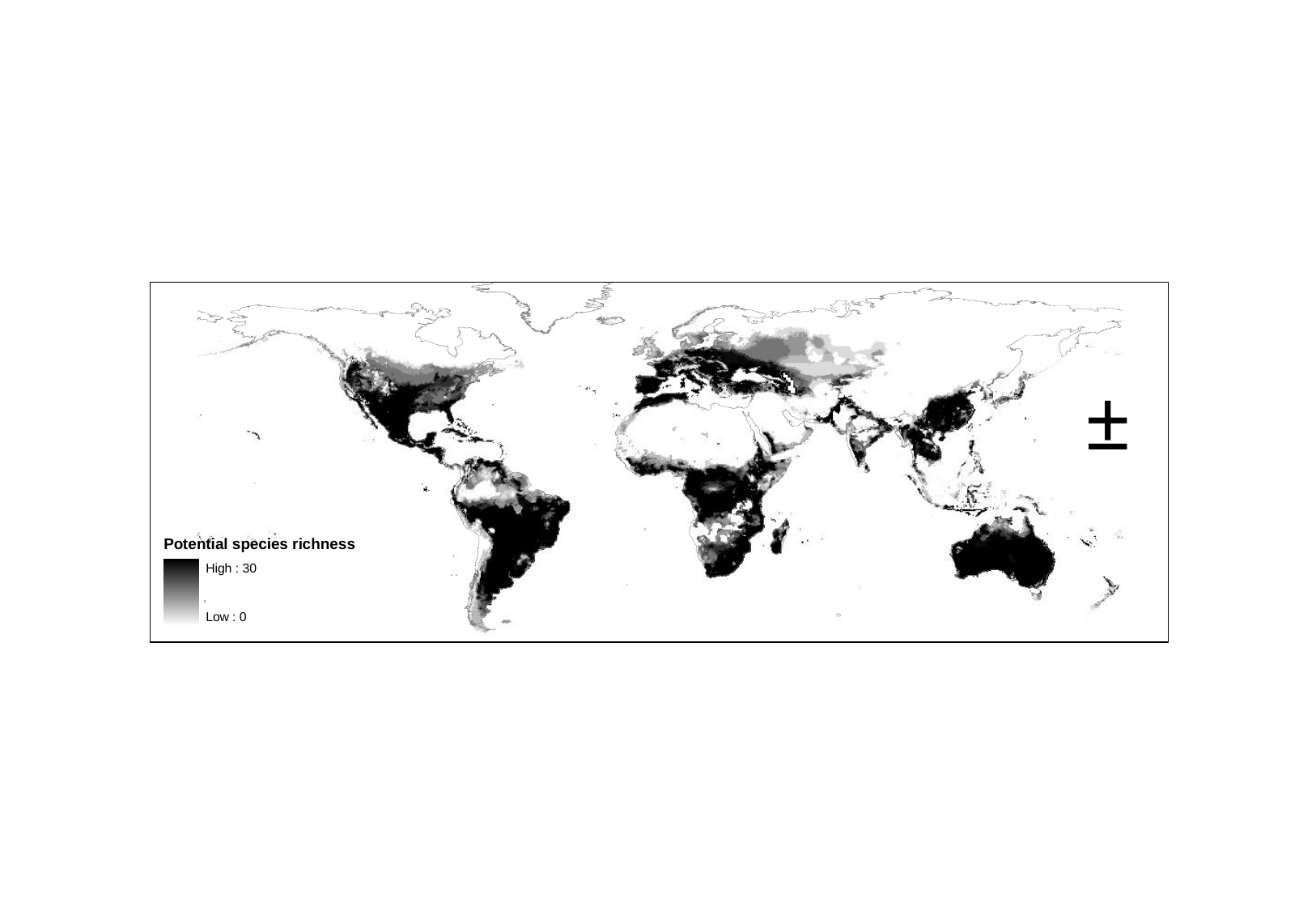

Supplement: Additional Information [file supp_plu078_plu078supp_file3.pptx]
