# Supplementary material for: Introduced and invasive cactus species: a global review
Source: AoB Plants. 2014 Dec 3;7:plu078. doi: 10.1093/aobpla/plu078 (PMC4318432; doi:10.1093/aobpla/plu078)
Supplement: Additional Information [file supp_plu078_plu078supp_file1_JW.docx]

Supporting information. File 1. Examples of sources of information on cactus species.

| Category | Source | Region/extent |
| --- | --- | --- |
| Alien species list | Weeds of National Significance (WoNS) | Australia |
| Alien species list | Decreto-Lei n.º 565/99 de 21 de Dezembro | Portugal |
| Alien species list | National Environmental Management: Biodiversity Act 2004 (Act No. 10 of 2004) Alien and Invasive Species Lists, 2014. Government Gazette of South Africa, vol. 559, issue 37886, pp. 3–80. Pretoria. | South Africa |
| Alien species list | Real Decreto 630/2013, de 2 de Agosto, por el que se regula el catalogo español de especies exóticas invasoras | Spain |
| Book | Anderson EF. 2001. *The cactus family*. Timber Press | Global |
| Book | Lloyd S, Reeves A. 2014. *Situation Statement on Opuntioid Cacti (Austrocylindropuntia spp ., Cylindropuntia spp . and Opuntia spp.) in Western Australia*. Department of Agriculture and Food. Goverment of Western Australia | Australia |
| Book | Walters M, Figueiredo E, Crouch NR, Winter PJD, Smith GF, Zimmermann HG, Mashope BK. 2011. *Naturalised and invasive succulents of southern Africa* (Y Samyn, D VandenSpiegel, and J Degreef, Eds.). Abc Taxa | South Africa |
| Book | Benson L. 1982. *The cacti of the United States and Canada*. Standford: Stanford University Press | United States and Canada |
| Cactus and succulents’ journal | Cactusvrieden | Belgium |
| Cactus and succulents’ journal | Terra seca | France |
| Cactus and succulents’ journal | Succulenta | Netherlands |
| Cactus and succulents’ journal | Cactus-adventures | Spain |
| Peer-review article | Ortega-Baes P, Aparicio-González M, Galíndez G, del Fueyo P, Sühring S, Rojas-Aréchiga M. 2010. Are cactus growth forms related to germination responses to light? A test using Echinopsis species. *Acta Oecologica* 36: 339–342 | Argentina |
| Peer-review article | Essl F, Kobler J. 2009. Spiny invaders – Patterns and determinants of cacti invasion in Europe. *Flora - Morphology, Distribution, Functional Ecology of Plants* 204: 485–494 | Europe |
| Peer-review article | Dean WR., Milton S. 2000. Directed dispersal of Opuntia species in the Karoo, South Africa: are crows the responsible agents? *Journal of Arid Environments* 45: 305–314 | South Africa |
| Peer-review article | Vilà M, Gimeno I. 2003. Seed predation of two alien Opuntia species invading Mediterranean communities. *Plant Ecology* 167: 1–8. | Spain |
| Online | <http://cactusguide.com/> | Global |
| Online | <http://cactus-art.biz/> | Global |
| Online | <http://www.environment.gov.au/cgi-bin/biodiversity/invasive/weeds> | Australia |
| Online | <http://www.invasives.org.za/> | South Africa |
